# Metabolic regulation of telomere silencing by SESAME complex-catalyzed H3T11 phosphorylation

**DOI:** 10.1038/s41467-020-20711-1

**Published:** 2021-01-26

**Authors:** Shihao Zhang, Xilan Yu, Yuan Zhang, Xiangyan Xue, Qi Yu, Zitong Zha, Madelaine Gogol, Jerry L. Workman, Shanshan Li

**Affiliations:** 1 State Key Laboratory of Biocatalysis and Enzyme Engineering, College of Life Sciences, Hubei University, Wuhan, Hubei 430062 China; 2 Human Aging Research Institute (HARI), School of Life Science, Nanchang University, Nanchang, Jiangxi 330031 China; 3 Stowers Institute for Medical Research, 1000 E. 50th Street, Kansas City, MO 64110 USA

**Keywords:** Multienzyme complexes, Autophagy, Gene silencing, Telomeres

## Abstract

Telomeres are organized into a heterochromatin structure and maintenance of silent heterochromatin is required for chromosome stability. How telomere heterochromatin is dynamically regulated in response to stimuli remains unknown. Pyruvate kinase Pyk1 forms a complex named SESAME (Serine-responsive SAM-containing Metabolic Enzyme complex) to regulate gene expression by phosphorylating histone H3T11 (H3pT11). Here, we identify a function of SESAME in regulating telomere heterochromatin structure. SESAME phosphorylates H3T11 at telomeres, which maintains SIR (silent information regulator) complex occupancy at telomeres and protects Sir2 from degradation by autophagy. Moreover, SESAME-catalyzed H3pT11 directly represses autophagy-related gene expression to further prevent autophagy-mediated Sir2 degradation. By promoting H3pT11, serine increases Sir2 protein levels and enhances telomere silencing. Loss of H3pT11 leads to reduced Sir2 and compromised telomere silencing during chronological aging. Together, our study provides insights into dynamic regulation of silent heterochromatin by histone modifications and autophagy in response to cell metabolism and aging.

## Abstract (web summary)

Pyruvate kinase phosphorylates histone H3T11 (H3pT11) and represses gene expression by forming a large complex SESAME (Serine-responsive SAM-containing Metabolic Enzyme). Here the authors show that SESAME-catalyzed H3pT11 regulates telomere silencing by promoting Sir2 binding at telomeres and preventing autophagy-mediated Sir2 degradation.

## Introduction

The eukaryotic genome is organized into distinct euchromatin and heterochromatin domains, which contain the bulk of actively transcribed regions and transcriptionally silent regions, respectively. Maintaining the integrity of euchromatin and heterochromatin within the nucleus is required for cell development and differentiation^1,2^. In budding yeast, heterochromatin is localized at three major sites, including silent mating-type loci (*HML* and *HMR*), telomeres, and ribosomal DNA (rDNA). Genes near these regions are subject to transcriptional repression, also known as position-effect variegation, which is highly conserved in higher organisms^3,4^. The proper organization of this transcriptionally silent heterochromatin is important to maintain telomere length, genome integrity, and chromosome segregation fidelity^5,6^. Disruption or loss of silent chromatin may lead to chromosomal instability, a hallmark and causal factor of tumorigenesis and cell aging^6–10^.

The silent heterochromatin at yeast telomeres is formed by assembly of Silent Information Regulator (SIR) complex (Sir2/Sir3/Sir4) and their spread along chromatin in a stepwise manner^11–13^. During this process, Sir4 is recruited by Rap1 and the yeast Ku complex to *cis*-acting silencer elements^14,15^. Sir4 then recruits Sir2, a highly conserved NAD^+^-dependent histone deacetylase (HDAC) to deacetylate H4K16^16^. Deacetylation of H4K16 generates a high-affinity binding site for Sir3, which then recruits more Sir4 and Sir2^17^. The cycles of Sir2-mediated H4K16 deacetylation and Sir3 recruitment allow the formation of SIR complex and its propagation along the telomere regions, which shields the telomere-proximal DNA from the transcription machinery, leading to transcription repression^4,11,18^. The spread of SIR complex to transcriptionally active chromatin is antagonized by H4K16ac, which acts as the heterochromatin–euchromatin boundary^19^. In addition to H4K16ac, Dot1-catalyzed H3K79me3 and Set1-catalyzed H3K4me3 have been shown to inhibit the spread of SIR complex into euchromatin regions^20,21^. These modifications occur predominantly in euchromatin regions and thus affect heterochromatin structure in an indirect manner^22^.

The silent heterochromatin is dynamically regulated in response to external and internal stimuli^2^. During the aging process, the global loss of heterochromatin was observed in *Drosophila*, *Caenorhabditis elegans*, and mammalian stem cells^6^. The telomere silencing is enhanced by increasing the temperature within the range of 23–37 °C in budding yeast^23^. Telomere silencing is also affected by cell metabolic states. During the aging process when carbon source is exhausted, the long-lived quiescent cells group their telomeres into a unique focus or hypercluster localized in the center of the nucleus, which correlates with more stable telomere silencing^24,25^. Changing carbon source from glucose to galactose induces telomere silencing defects, whereas switching from glucose to glycerol enhances telomere silencing by affecting the redox status^26,27^. Calorie restriction (CR) by glucose depletion has been proposed to increase NAD^+^/NADH and enhance Sir2 activity, which promotes telomere silencing^28^. However, glucose deficiency has also been shown to have no significant effect on Sir2 activity and telomere silencing^29,30^. These results suggest the relationship between glucose metabolism and telomere silencing is complex.

Some metabolic enzymes have been reported to translocate into the nucleus, where they regulate telomere silencing. For example, the glyceraldehyde 3-phosphate dehydrogenase (Tdh3) enhances telomere silencing by maintaining the nuclear NAD^+^ levels, suggesting that it is nuclear NAD^+^ instead of global NAD^+^ changes that regulate telomere silencing^31^. The glutamate dehydrogenase 1 (Gdh1) can translocate into the nucleus and maintain telomere silencing likely by reducing nuclear α-ketoglutarate levels^32^. All these results emphasize the importance of examining the nuclear functions of metabolic enzymes in modulating telomere silencing.

We have previously found that the glycolytic enzyme, pyruvate kinase (Pyk1), can translocate into the nucleus, where it exerts non-metabolic functions. Pyk1 phosphorylates histone H3T11 (H3pT11) to repress the expression of a subset of genes as a part of a 400 kDa complex, Serine-responsive SAM-containing Metabolic Enzyme (SESAME), which contains serine metabolic enzymes, SAM synthetases, and an acetyl-CoA synthetase^33^. Glycolysis and serine metabolism stimulate SESAME to phosphorylate H3T11 and confer cell resistance to oxidative stress^33,34^. In this study, by examining the genome-wide binding profiles for SESAME and H3pT11, we unexpectedly discovered that SESAME binds at telomeres to phosphorylate H3T11. SESAME-catalyzed H3pT11 maintains telomere silencing, promotes SIR complex binding at telomeres, and prevents autophagy-mediated Sir2 degradation. Our study also reveals a dynamic regulation of silent heterochromatin assembly at telomeres by SESAME-phosphorylated H3T11 in response to cell metabolism and aging.

## Results

### H3T11 phosphorylation is enriched at gene promoters and telomere-proximal regions

**Fig. 1:**
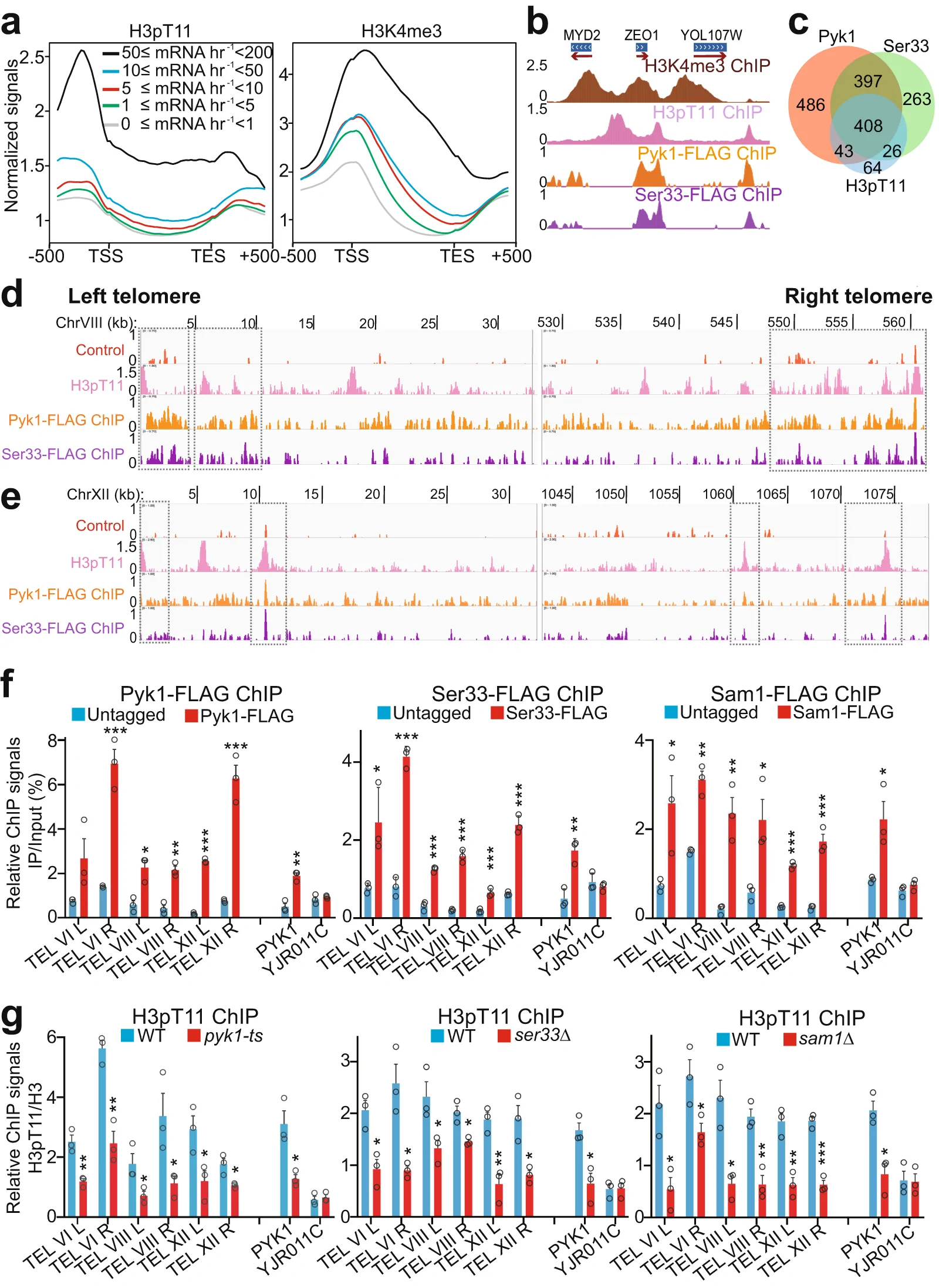
SESAME phosphorylates H3T11 at gene promoters and telomere regions. **a** The composite binding profile of H3pT11 at 5065 genes which were divided into five groups according to their transcription rates. The log_2_ ratio of normalized enrichment (H3pT11/H3, H3K4me3/H3) for each gene region, including 500 bp upstream and downstream regions from the gene, were used for average gene analysis. The transcription start site (TSS) and termination site (TES) are indicated. **b** ChIP-seq tracks showing the occupancy of H3K4me3/H3, H3pT11/H3, Pyk1, and Ser33 at representative genes. **c** Venn diagram of genes that overlap among the Pyk1-peak, Ser33-peak, and H3pT11-peak gene sets as determined by ChIP-seq. The numbers of binding sites are represented. **d**, **e** ChIP-seq tracks showing the occupancy of H3pT11/H3, Pyk1, and Ser33 at the left and right telomere regions of chromosome VIII (TEL VIII) (**d**) and chromosome XII (TEL XII) (**e**). The control tracks were generated using untagged BY4741 strain with anti-FLAG antibody. **f** ChIP-qPCR analysis of Pyk1, Ser33, and Sam1 at telomeres of chromosome VI (TEL VIL and TEL VIR), chromosome VIII (TEL VIIIL and TEL VIIIR), and chromosome XII (TEL XIIL and TEL XIIR). The *YJR011C* was used as a non-target region. **g** ChIP-qPCR analysis of H3pT11 at telomeres in WT, *pyk1-ts*, *ser33Δ*, and *sam1Δ* mutants. Pyk1 was inactivated by growing *pyk1-ts* mutant at non-permissive temperature (37 °C) for 2 h. H3pT11 was normalized to histone H3 level. For Fig. 1f, g, the quantitative data represent the mean ± SE; n = 3 biological independent experiments. Statistical significance was tested using two-sided Student’s *t* test. *p < 0.05; **p < 0.01; ***p < 0.001.

To determine the genome-wide localization of H3T11 phosphorylation (H3pT11), we performed chromatin immunoprecipitation followed by high-throughput sequencing (ChIP-seq) of H3pT11 in yeast cells grown in glucose-containing rich media and compared it with the positions of H3K4me3, a histone marker associated with active gene transcription. Figure 1a shows a metagene profile and Fig. 1b shows a browser track of representative genes. These data illustrate that H3pT11 is highly enriched upstream of the transcription start site (TSS) and flanks the peaks of H3K4me3 (Fig. 1a, b), consistent with the localization of H3pT11 at the *PYK1* gene promoter^33^. We also classified RNA polymerase II-regulated genes into five groups based on their transcription levels and then compared H3pT11 levels among these groups. The H3pT11 signals were positively correlated with gene transcription levels (Fig. 1a). Like the occurrence of repressive histone markers, H3K36 methylation and H3K9 methylation at active gene regions^35–37^, the enrichment of H3pT11 at highly expressed genes may fine-tune their expression. Moreover, H3pT11 was enriched at 541 genes, among which 408 genes were co-occupied by SESAME subunits, Pyk1 and Ser33 (Fig. 1c). Kyoto Encyclopedia of Genes and Genomes (KEGG) pathway analysis revealed that these 408 genes were involved in pathways such as ribosome, metabolism, longevity regulating pathways, phagosome, autophagy, protein export, etc. (Supplementary Fig. 1a).

Interestingly, we noticed a significant number of peaks for H3pT11 in the vicinity of the telomeres (unique subtelomeric sequences) (Fig. 1d, e and Supplementary Fig. 1b). We also observed the binding of SESAME subunits, Pyk1 and Ser33 at these regions (Fig. 1d, e and Supplementary Fig. 1b). The occupancy of Pyk1, Ser33, and Sam1 at subtelomere regions was confirmed by ChIP followed by quantitative real-time PCR (qPCR) (Fig. 1f). Compared to non-target gene *YJR011C*, there is a 2.01-, 2.16-, 2.95-fold enrichment of Pyk1, Ser33, and Sam1 at the non-telomeric *PYK1* gene (Fig. 1f). Compared to *YJR011C*, there is a 2.29–7.38-fold, 1.55–5.15-fold, 1.57–4.13-fold enrichment of Pyk1, Ser33, and Sam1 at telomere regions (Fig. 1f), suggesting that SESAME has similar occupancy at telomere regions and euchromatin target regions. The localization of H3pT11 at these regions was also confirmed by ChIP-qPCR (Fig. 1g). The H3pT11/H3 ChIP signal at the *PYK1* gene was 2.81–5.37-fold higher than that at *YJR011C* (Fig. 1g). The H3pT11/H3 ChIP signals at telomere regions was 2.53–9.71-fold higher than that at *YJR011C* (Fig. 1g). The variability of SESAME and H3pT11 occupancy at telomeres could be due to genetic diversity and irregularity of native repressed chromatin at telomeres^38^. Moreover, the enrichment of H3pT11 at these regions was significantly reduced in SESAME mutants, including *pyk1-ts*, *ser33Δ*, and *sam1Δ* mutants (Fig. 1g). The loss of H3pT11 occupancy at different telomere regions is variable, which could be caused by the different binding affinity of SESAME complex at different regions and Pyk1 is partially inactivated at 37 °C. These results indicate that the SESAME complex phosphorylates H3T11 at telomere-proximal regions.

Set1 and H3K4me3 are required to recruit SESAME to phosphorylate H3T11 at active genes, i.e., *PYK1* and *PMA1*^33^. We then examined the effect of Set1 and H3K4me3 on the occupancy of SESAME and H3pT11 at telomeres. In *set1Δ* and H3K4A mutants, H3pT11 occupancy at the *PYK1* gene was significantly reduced but its occupancy at telomeres was unaffected (Supplementary Fig. 1c). Similarly, loss of Set1 did not reduce the occupancy of SESAME complex at telomeres despite reduced SESAME enrichment at the *PYK1* gene (Supplementary Fig. 1d), indicating that the binding of SESAME at telomeres is independent of Set1-catalyzed H3K4me3.

### SESAME-catalyzed H3pT11 maintains telomere-specific transcription silencing

**Fig. 2:**
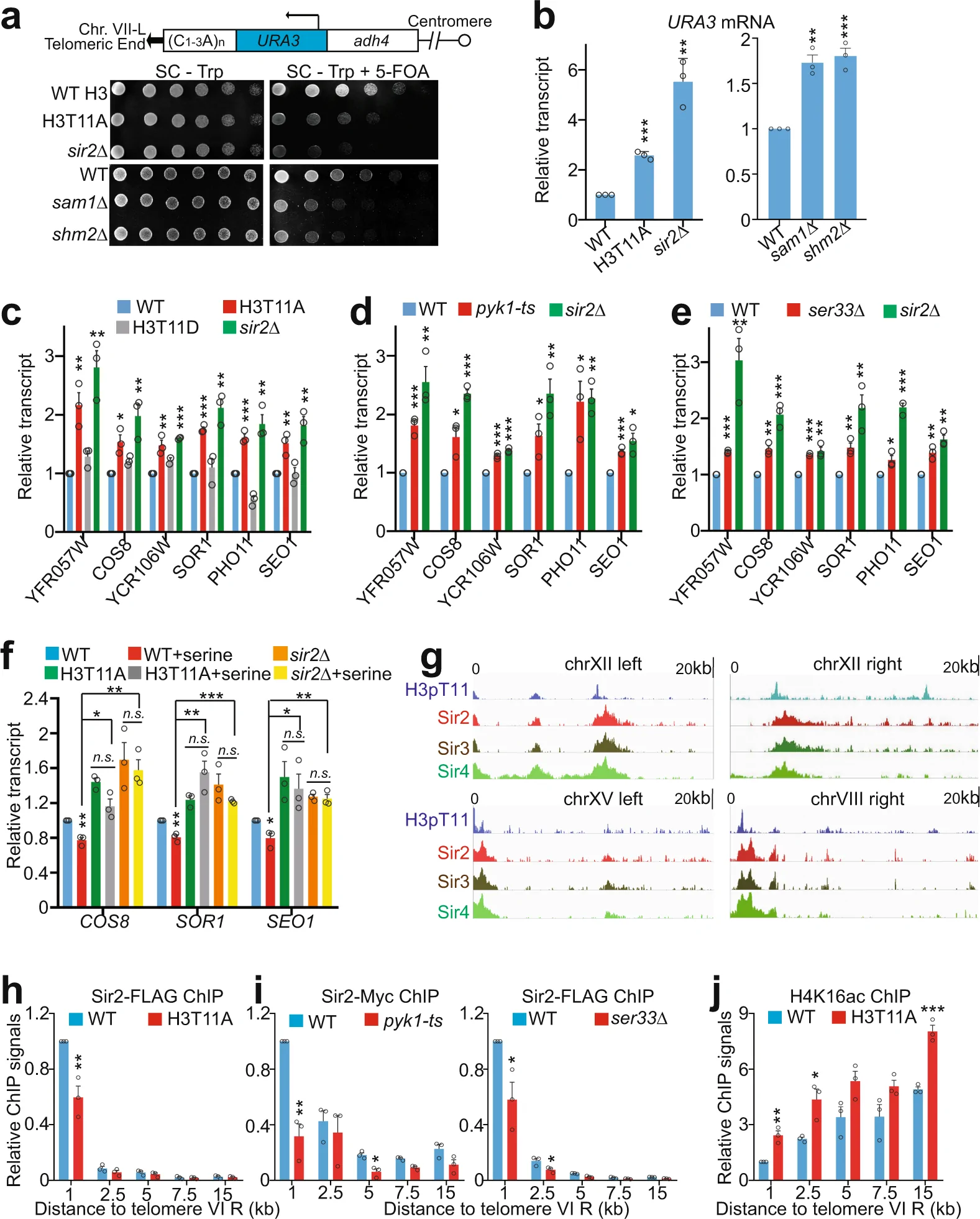
SESAME-catalyzed H3pT11 promotes SIR complex binding at telomeres and is required to maintain normal telomere silencing. **a** SESAME-catalyzed H3pT11 is required to maintain telomere silencing. Top panel: Diagram of the position of *URA3* inserted near the telomere VII-L in the telomere silencing reporter strain. Bottom panel: WT, H3T11A, *sir*2Δ, *sam1Δ*, and *shm2Δ* cells bearing *URA3* adjacent to Tel VII-L were grown to saturation, normalized for OD_600_, 3-fold serially diluted and spotted on SC - Trp and SC - Trp + 5-FOA plates. Impaired growth on 5-FOA plates indicates diminished silencing of *URA3*. **b** qRT-PCR analysis of *URA3* in WT, H3T11A, *sir*2*Δ*, *sam1Δ*, and *shm2Δ* mutants. **c**–**e** qRT-PCR analysis of native telomere-proximal genes (*YFR057W*, *COS8*, *YCR106W*, *SOR1*, *SEO1*) in WT, H3T11A (**c**), H3T11D (**c**), *pyk1-ts* (**d**), *ser33Δ* (**e**), and *sir*2*Δ* mutants. **f** Effect of serine on the transcription of native telomere-proximal genes (*COS8*, *SOR1*, and *SEO1*) in WT, H3T11A, and *sir*2*Δ* mutants as determined by qRT-PCR analysis. **g** ChIP-seq tracks showing the enrichment of H3pT11/H3, Sir2, Sir3, and Sir4 at regions within 20 kb of representative telomeres. **h**, **i** ChIP analysis of Sir2 occupancy at regions with different distance (1, 2.5, 5, 7.5, and 15 kb) to telomere VI-R in WT, H3T11A (**h**), *pyk1-ts* (**i**), and *ser33Δ* (**i**) mutants. **j** ChIP analysis of H4K16ac at regions with different distance (1, 2.5, 5, 7.5, and 15 kb) to telomere VI-R in WT and H3T11A mutant. H4K16 acetylation was normalized to its input. For Fig. 2b–f, h–j, the quantitative data represent the mean ± SE; *n* = 3 biological independent experiments. Statistical significance was tested using two-sided Student’s *t* test. *p < 0.05; **p < 0.01; ***p < 0.001. For Fig. 2a, shown are the typical example of three biological independent biological replicates.

To examine the effect of SESAME-catalyzed H3pT11 on telomere silencing, the H3T11A mutation was introduced into a telomere silencing reporter strain, which has the *URA3* reporter gene inserted adjacent to the left telomere of chromosome VII (Tel VII-L) (Fig. 2a)^39^. In this reporter assay, telomere silencing is reflected by cell growth on medium containing 5-fluoroorotic acid (5-FOA), a suicide substrate for cells expressing *URA3*. The growth of H3T11A mutant on 5-FOA plate was reduced when compared to its wild-type counterpart cells (Fig. 2a). Similar impaired growth on 5-FOA plate was also observed in SESAME mutants, *sam1Δ* and *shm2Δ* (Fig. 2a). The reduced growth of H3T11A, *sam1Δ*, and *shm2Δ* mutants on 5-FOA plates was caused by increased transcription of *URA3* as determined by quantitative reverse transcription PCR (qRT-PCR) (Fig. 2b), indicating telomere silencing defects in SESAME and H3T11A mutants. We further confirmed this result in another telomere silencing reporter strain, harboring the *ADE2* reporter gene inserted adjacent to the right telomere of chromosome V (Tel V-R). Cells with normal silenced *ADE2* are red after grown for 4–6 days but will become white once *ADE2* is derepressed^39^. In agreement with the results with the *URA3* reporter assay, we observed that H3T11A mutant cells became white (Supplementary Fig. 2a) and this color change was due to increased *ADE2* expression in the H3T11A mutant (Supplementary Fig. 2b).

In addition to telomere silencing reporter assays, we examined the effect of H3pT11 on the transcription of naturally occurring telomere-linked genes by qRT-PCR. The typical example is *YFR057W*, which is located near the right telomere of chromosome VI (Tel VI-R) and subject to transcriptional silencing in a SIR-dependent manner (Fig. 2c). *YFR057W* was upregulated in H3T11A mutant but not in H3T11D mutant, which mimics H3T11 phosphorylation (Fig. 2c). We also investigated the transcription of genes near other telomeres, including *COS8*, *YCR106W*, *SOR1*, *PHO11*, and *SEO1*, which locate 6.4 kb from Tel VIII-L, 3.2 kb from Tel III-R, 8.6 kb from Tel X-R, 3.4 kb from Tel I-L, and 7.2 kb from Tel I-L, respectively. Similar to *YFR057W* derepression, the transcription of these genes was increased in H3T11A but not in H3T11D mutant (Fig. 2c), indicating that the transcriptional silencing defects occur at multiple telomeres and is gene independent. Similar derepression of these genes was observed in *pyk1-ts*, *ser33Δ*, *sam1Δ*, and *shm2Δ* mutants (Fig. 2d, e and Supplementary Fig. 2c). Moreover, deletion of *SER33* or *SHM2* in H3T11A mutant did not further increase the transcription of telomere-proximal genes when compared with H3T11A mutant (Supplementary Fig. 2d). These two independent assays indicate that SESAME-catalyzed phosphorylation of H3T11 is required for maintenance of natural telomere silencing.

Serine has been shown to stimulate SESAME complex to phosphorylate H3T11^33^. Indeed, serine treatment significantly increased the global levels of H3pT11 (Supplementary Fig. 2e). We thus examined the effect of serine on telomere silencing. Serine but not glycine treatment repressed the transcription of genes near the telomeres (Supplementary Fig. 2f) and this enhanced silencing effect by serine was not observed in the H3T11A and *sir2Δ* mutants (Fig. 2f), indicating that H3pT11 is required for serine to enhance telomere silencing.

We also examined the effect of SESAME-catalyzed H3pT11 on transcriptional silencing at *HM* loci by the reporter assay with *URA3* integrated near the silent mating loci, *HML* or *HMR*. The SESAME mutants showed no detectable defects in *HM* silencing (Supplementary Fig. 2g, h), suggesting that SESAME-phosphorylated H3T11 represses transcription of genes near telomeres but not *HM* loci. The ChIP-seq data for H3pT11 also showed very low signals of H3pT11 at *HM* loci (Supplementary Fig. 2i). This locus-specific silencing effect has also been observed in the mutants of the Mediator complex and H3K79 methyltransferase Dot1, which have reduced natural telomere silencing but intact *HM* silencing^40,41^.

### Loss of SESAME-catalyzed H3pT11 leads to reduced occupancy of SIR complex at telomere regions

By comparing our ChIP-seq data of H3pT11 to those of known telomere regulating proteins, we found that H3pT11 has a similar distribution pattern at telomere regions with the SIR (Sir2/Sir3/Sir4) complex (Fig. 2g and Supplementary Fig. 3a), suggesting that H3pT11 could be functionally related to the SIR complex in regulation of telomere silencing.

We first examined whether H3pT11 is dependent on SIR complex. Depletion of SIR proteins had no significant effect on global H3pT11 levels as well as H3pT11 at telomere regions (Supplementary Fig. 3b, c). We then examined the effect of H3T11A mutation on SIR binding at telomeres by ChIP-qPCR. Loss of H3pT11 led to reduced Sir2 occupancy at telomeres (Tel VI, Tel VIII, Tel XII) (Fig. 2h and Supplementary Fig. 3d). The Sir2 levels were also significantly reduced at telomere-proximal genes in H3T11A mutant (Supplementary Fig. 3e), consistent with their derepression in H3T11A mutant (Fig. 2c). In contrast, loss of H3pT11 had no significant effect on Sir2 binding at *HML* and rDNA loci (Supplementary Fig. 3f), consistent with no significant effect of SESAME on *HM* loci silencing (Supplementary Fig. 2g, h). Similar reduced Sir2 occupancy at telomeres and telomere-proximal genes was observed in *pyk1-ts* and *ser33Δ* mutants (Fig. 2i and Supplementary Fig. 3d, e). As Sir2 specifically deacetylates H4K16 at telomere proximity regions^42^, we thus asked whether loss of H3pT11 would affect H4K16ac as a result of Sir2 dissociation from telomeres. Indeed, H4K16ac was significantly increased at these regions in H3T11A mutant (Fig. 2j), consistent with reduced Sir2 occupancy in H3T11A mutant (Fig. 2h). In addition, the occupancy of Sir3 and Sir4 at telomeres was significantly reduced in H3T11A mutant (Supplementary Fig. 3g, h), suggesting that H3pT11 promotes SIR complex binding at telomeres.

### SESAME-catalyzed H3pT11 is required to maintain normal Sir2 protein levels

**Fig. 3:**
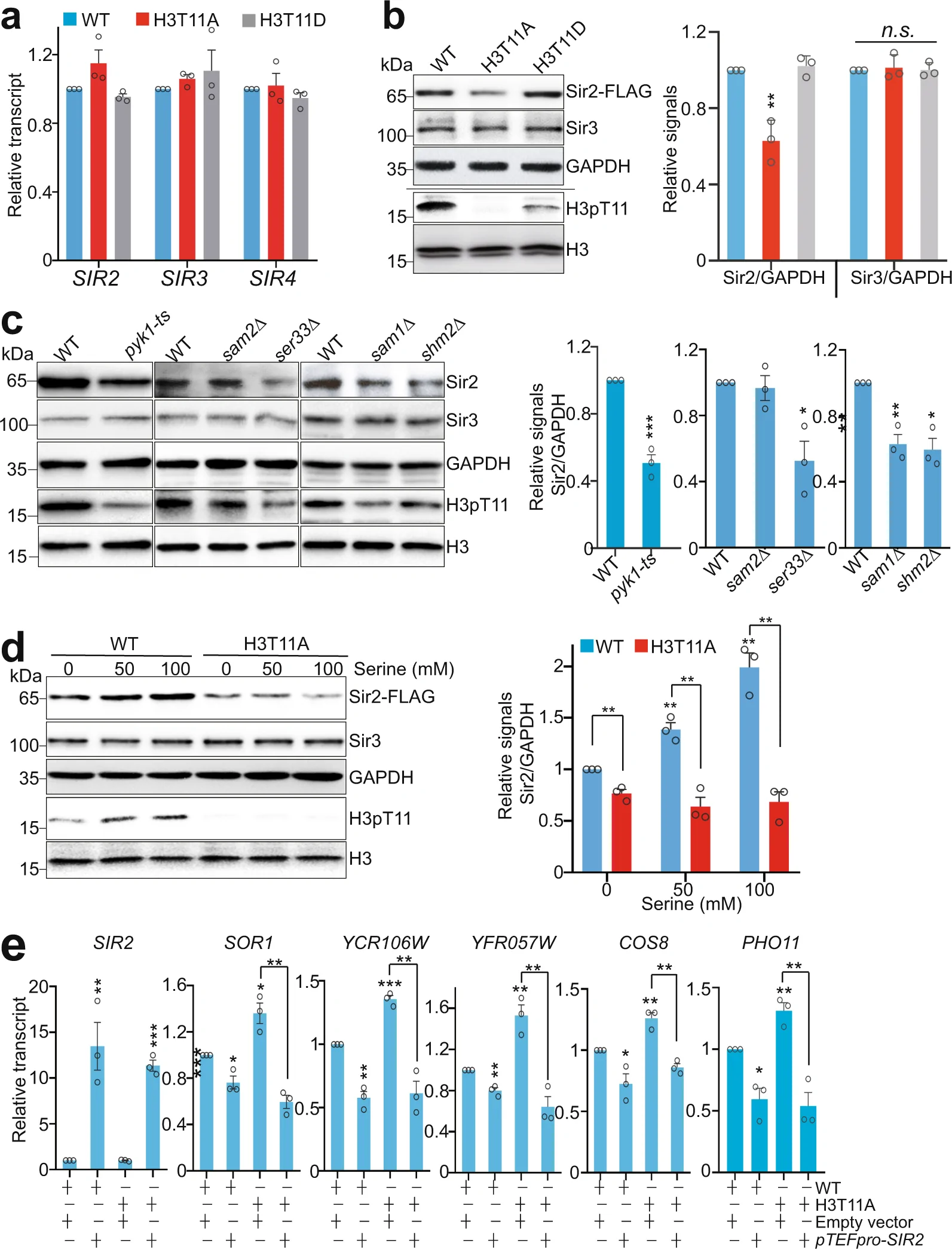
SESAME-catalyzed H3pT11 is required to maintain global Sir2 protein levels. **a** qRT-PCR analysis of *SIR2*, *SIR3*, and *SIR4* expression in WT, H3T11A, and H3T11D mutants. **b** Left panel: Western blots analysis of Sir2 and Sir3 in WT, H3T11A, and H3T11D mutants. The H3pT11 antibody can recognize the phosphomimic site (aspartate) at H3T11D with low efficiency. Right panel: The relative intensities of Sir2/GAPDH and Sir3/GAPDH in left panel were quantified using ImageJ with standard error (SE). Data represent the mean ± SE of three biological independent experiments. **c** Sir2 protein levels were significantly reduced in WT, *pyk1-ts*, *ser33Δ, sam1Δ*, and *shm2Δ* mutants with the exception of *sam2Δ* as determined by Western blots analysis. **d** Serine significantly increased the global Sir2 protein levels in WT but not H3T11A mutant. **e** qRT-PCR analysis of *SIR2*, *SOR1*, *YCR106W*, *YFR057W*, *COS8*, and *PHO11* in WT and H3T11A mutant transformed with empty vector or vector that overexpresses Sir2 (*pTEFpro-SIR2*). For Fig. 3a–e, the quantitative data represent means ± SE; *n* = 3 biological independent experiments. Statistical significance was tested using two-sided Student’s *t* test. *p < 0.05; **p < 0.01; ***p < 0.001; n.s. no significance.

We then examined the effect of H3pT11 on the expression of SIR proteins. Although the transcription of *SIR2*, *SIR3*, and *SIR4* was not significantly changed in H3T11A and SESAME mutants (Fig. 3a and Supplementary Fig. 4a), Sir2 protein levels were significantly reduced in H3T11A mutant but not in H3T11D mutant (Fig. 3b). Sir2 protein levels were also significantly reduced in H3T11A mutant in different strain backgrounds (Supplementary Fig. 4b). In contrast, Sir3 and Sir4 were not significantly changed in H3T11A mutant (Fig. 3b and Supplementary Fig. 4c).

To confirm it is SESAME-catalyzed H3pT11 that regulates the steady-state level of Sir2, we examined Sir2 protein levels in SESAME mutants, including *pyk1-ts*, *sam1Δ*, *sam2Δ*, *ser33Δ*, and *shm2Δ*. Similar to the H3T11A mutant, Sir2 but not Sir3 was significantly reduced in these mutants with the exception of *sam2Δ* (Fig. 3c). The reduced Sir2 in *sam1Δ* mutant but not *sam2Δ* mutant could be caused by the fact that their expression is regulated differently^43^. As serine stimulates SESAME complex to phosphorylate H3T11 and enhance telomere silencing (Fig. 2f and Supplementary Fig. 2e), we also examined the effect of serine on Sir2 protein levels. Serine treatment significantly increased the global levels of H3pT11 and Sir2 (Supplementary Fig. 4d). As a control, glycine did not increase H3pT11 and Sir2 protein levels (Supplementary Fig. 4d). Moreover, this stimulatory effect of serine on Sir2 protein levels was observed in WT but not in H3T11A mutant (Fig. 3d), consistent with the enhanced effect of serine on telomere silencing in WT but not in H3T11A mutant (Fig. 2f).

To examine whether SESAME-catalyzed H3pT11 regulates telomere silencing by maintaining Sir2 protein levels, we examined whether overexpression of Sir2 can rescue the telomere silencing defects in H3T11A mutant. We transformed WT and H3T11A mutant with the construct that overexpresses Sir2 under the constitutive strong *TEF1* promoter (*pTEFpro-SIR2*). The transcription of telomere proximity genes was significantly increased in H3T11A mutant; however, overexpression of Sir2 abrogated the increased transcription of these genes in H3T11A mutant (Fig. 3e), suggesting that the telomere silencing defects in H3T11A mutant can be rescued by Sir2 overexpression. Together, these results indicate that SESAME-catalyzed H3pT11 is required to maintain Sir2 homeostasis and telomere silencing.

### Loss of H3pT11 leads to Sir2 degradation by autophagy

**Fig. 4:**
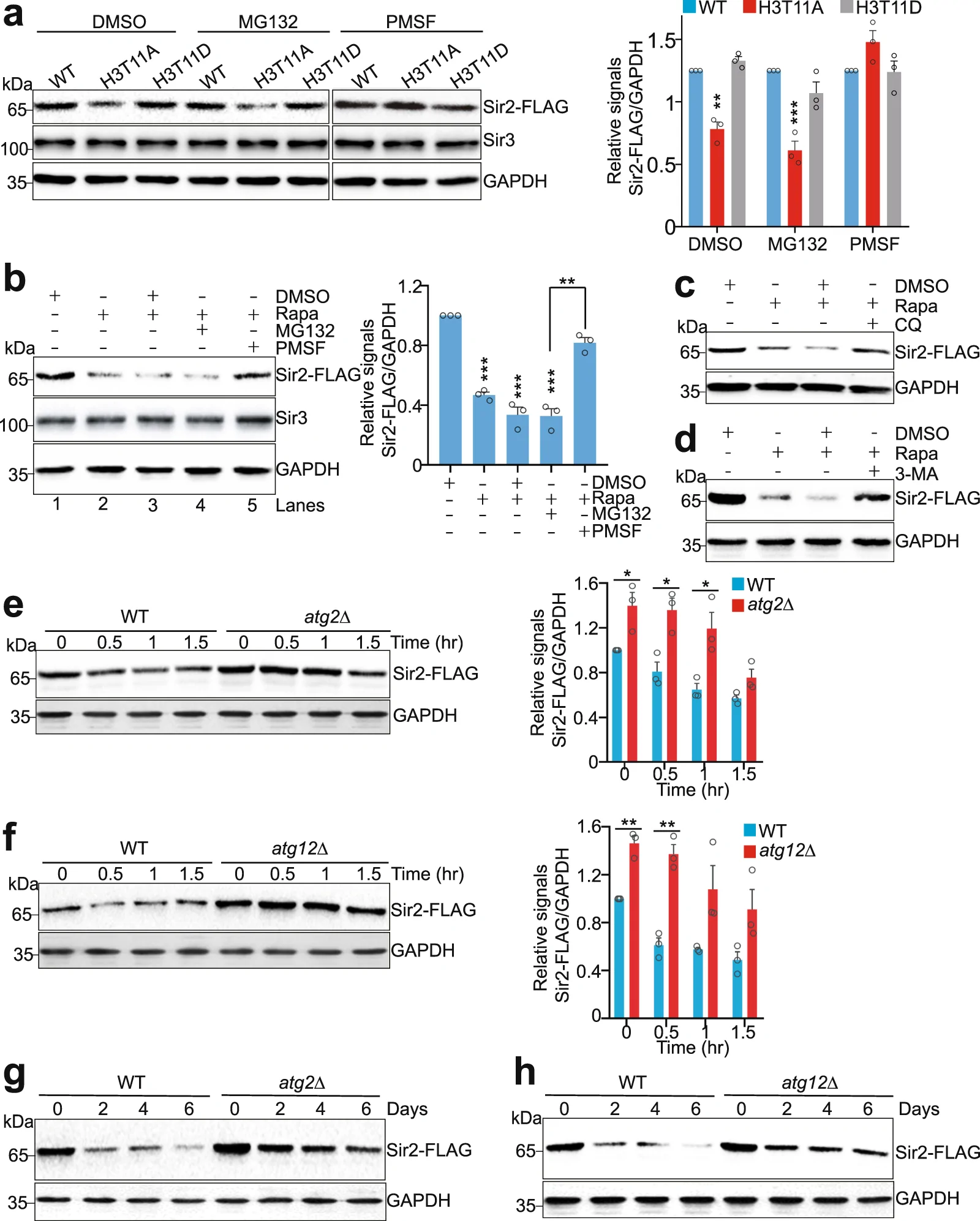
Sir2 is degraded by the autophagy pathway. **a** Western blots showing PMSF but not MG132 treatment rescued the reduced Sir2 proteins in the H3T11A mutant. The endogenously expressed Sir2-FLAG and Sir3 in WT, H3T11A, and H3T11D mutants were examined with anti-FLAG and anti-Sir3 antibodies, respectively. **b** Rapamycin-reduced Sir2 protein levels were partially rescued by PMSF treatment as determined by Western blots. Rapa rapamycin. **c**, **d** Representative Western blots showing rapamycin-reduced Sir2 protein levels were partially rescued by chloroquine (CQ) and 3-MA. **e**, **f** Western blots showing the global Sir2 protein levels were significantly higher in *atg2Δ* (**e**) and *atg12Δ* (**f**) mutants than in WT when treated with rapamycin. WT, *atg2Δ*, and *atg12Δ* mutants were treated with 1 μg/ml rapamycin for 0–1.5 h. **g**, **h** The global Sir2 protein levels were significantly higher in *atg2Δ* and *atg12Δ* mutants than in WT when aged for 0–6 days in YPD medium as determined by Western blots. For Fig. 4a, b, e, f, the quantitative data represent means ± SE; *n* = 3 biological independent experiments. Statistical significance was tested using two-sided Student’s *t* test. *p < 0.05; **p < 0.01; ***p < 0.001. For Fig. 4c, d, g, h, shown are the typical example of three biological independent experiments.

In eukaryotic cells, there are two major protein degradation systems to regulate protein stability: the 26S proteasome and the lysosome pathways^44^. To identify the mechanism(s) responsible for Sir2 degradation in H3T11A mutant, we treated WT and H3T11A cells with the proteasome inhibitor, MG132 and found that the reduced Sir2 in H3T11A mutant was unaffected by MG132 treatment (Fig. 4a), suggesting that Sir2 protein in H3T11A mutant was degraded independent on the proteasome pathway.

Autophagy is a lysosome-dependent process that degrades cell components, proteins, and damaged organelles to maintain cell homeostasis^45^. Autophagy includes a stepwise process including autophagosome formation, fusion of the autophagosome with a lysosome, and cargos degradation, which requires a set of autophagy-related (ATG) proteins that coordinately function at different stages^46,47^. To test whether autophagy regulates Sir2 degradation, we treated cells with Phenylmethanesulfonyl fluoride (PMSF), an inhibitor for lysosomal proteases^48^. The reduced Sir2 proteins in H3T11A mutant were restored by PMSF treatment (Fig. 4a). When cells were treated with rapamycin (Rapa), an mTOR kinase inhibitor to induce autophagy, the Sir2 protein levels were significantly reduced (Fig. 4b, lane 2 vs lane 1). As a control, Sir3 and Sir4 were not degraded by rapamycin treatment (Supplementary Fig. 5a–c). Rapamycin-induced Sir2 degradation was ameliorated by adding PMSF but not MG132 (Fig. 4b, lane 4 vs lane 5). We also treated cells with rapamycin along with autophagy inhibitors, chloroquine (CQ) and 3-methyladenine (3-MA) and found that both CQ and 3-MA can prevent rapamycin-induced Sir2 degradation (Fig. 4c, d). These data further support that Sir2 is degraded by autophagy.

To determine whether autophagy proteins regulate Sir2 degradation, we examined the global Sir2 protein levels in autophagy gene deletion mutants in the presence or absence of rapamycin treatment (Supplementary Fig. 5d). In particular, deletion of *ATG2* and *ATG12* significantly inhibited rapamycin-induced Sir2 degradation (Fig. 4e, f), whereas no rescue effect was observed in *atg5Δ*, *atg7Δ*, and *atg9Δ* mutants (Supplementary Fig. 5d), suggesting that different autophagy pathways could be responsible for Sir2 degradation.

The above data suggest that Sir2 is degraded by the short-term (0–1.5 h) autophagy process. As heterochromatin is gradually lost during aging^6^ and SESAME-catalyzed H3pT11 is enriched in genes regulating longevity pathways (Supplementary Fig. 1a), we thus examined the effect of aging-induced long-term autophagy on Sir2 status (Supplementary Fig. 5e). When cells were aged for 0, 2, 4, and 6 days, Sir2 protein levels were significantly reduced during chronological aging (Fig. 4g). Notably, the overall levels of Sir2 in *atg2Δ* and *atg12Δ* mutants were higher than those in WT during aging process (Fig. 4g, h), suggesting that Sir2 is degraded by autophagy during chronological aging.

### SESAME-catalyzed H3pT11 prevents Sir2 nuclear export and inhibits autophagy-mediated Sir2 degradation

**Fig. 5:**
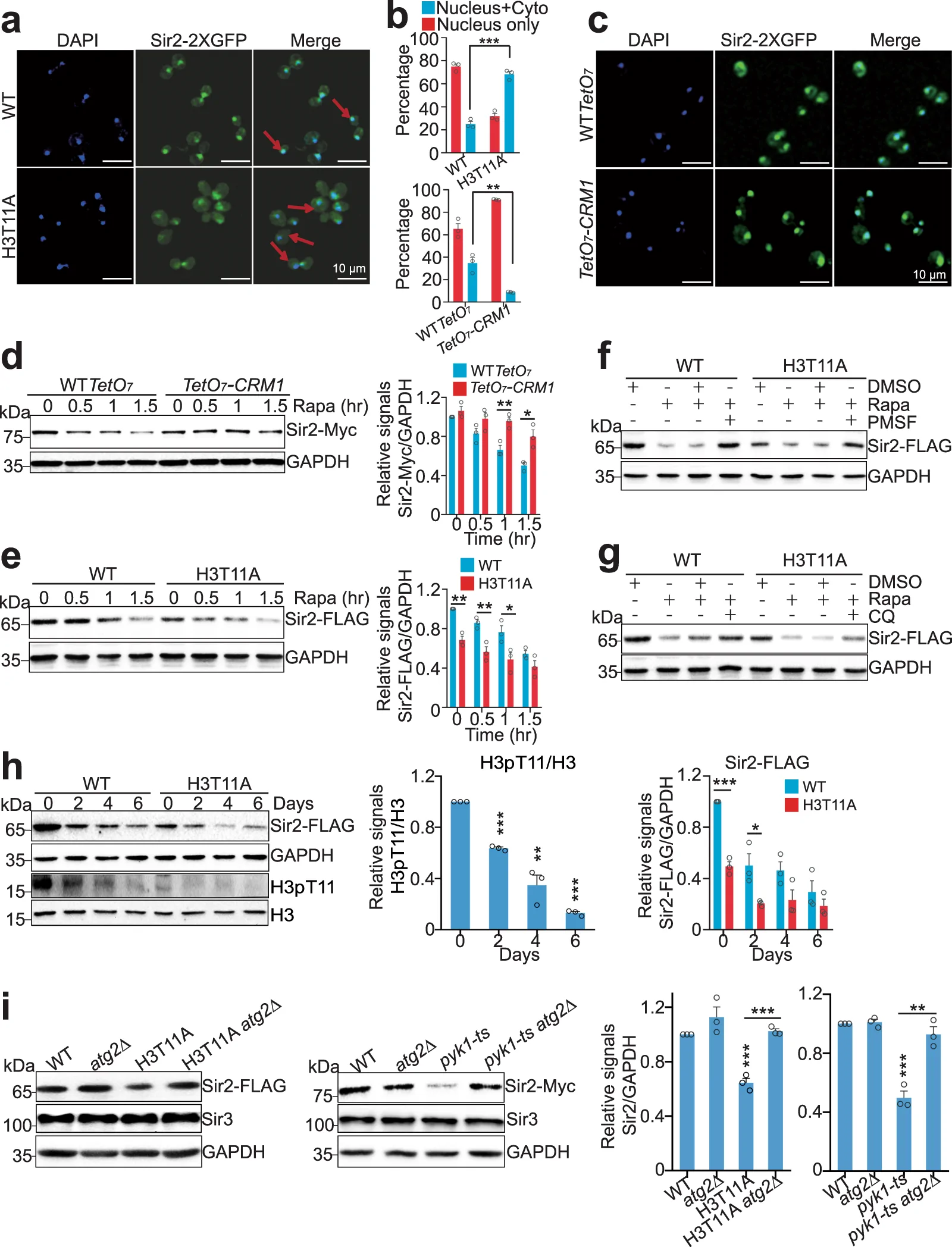
SESAME-catalyzed H3pT11 prevent the nuclear export of Sir2 and autophagy-mediated Sir2 degradation. **a** Representative fluorescence images showing the distribution of Sir2-2xGFP (green) in WT and H3T11A cells expressing Sir2-2xGFP from the native *SIR2* locus. The nucleus DNA was stained with 4’,6-diamidino-2-phenylindole (DAPI) as shown in blue. There was more Sir2 diffuse throughout the cell in H3T11A mutant compared with WT. Arrows indicate the nucleus-localized Sir2 in WT and cytoplasm-localized Sir2 H3T11A mutant, respectively. Bar, 10 μm. **b** Quantification of Sir2-2xGFP localization in cells displayed in Fig. 5a, c, respectively. The bar graphs represent the percentages of cells exhibiting Sir2-2xGFP localized in the nucleus (Nucleus only), or exported to the cytoplasm (Nucleus + Cyto). Data show mean ± SE from at least three biological independent experiments, with ∼350 cells counted for each strain per experiment. **c** Knockdown of *CRM1* expression retained most Sir2 in the nucleus. WT *TetO*_*7*_ and *TetO*_*7*_*-CRM1* cells grown in YPD medium were treated with 6.25 μg/ml doxycycline for 1.5 h followed by fluorescence microscopy. Bar, 10 μm. **d** Knockdown of *CRM1* expression partly rescued rapamycin-reduced Sir2. WT *TetO*_*7*_ and *TetO*_*7*_*-CRM1* cells grown in YPD medium were treated with 6.25 μg/ml doxycycline and rapamycin for 0–1.5 h. **e** The global Sir2 protein levels in H3T11A mutant were significantly lower than those in WT when treated with rapamycin. **f**, **g** Rapamycin-reduced Sir2 in H3T11A mutant was partly rescued by PMSF (**f**) and CQ (**g**) treatments. **h** Western blots analysis of Sir2 and H3pT11 in WT and H3T11A mutant when aged for 0–6 days in YPD medium. **i** Western blots showing deletion of *ATG2* rescued the reduced Sir2 in H3T11A and *pyk1-ts* mutants. For WT, *atg2Δ*, *pyk1-ts*, and *pyk1-ts atg2Δ* mutants, these four strains were treated at 37 °C for 2 h. For Fig. 5b, d, e, h, i, the quantitative data represent means ± SE; *n* = 3 biological independent experiments. Statistical significance was tested using two-sided Student’s *t* test. *p < 0.05; **p < 0.01; ***p < 0.001. For Fig. 5f, g, shown are the typical example of three biological independent experiments.

Sir2 is known as a silencing protein in the nucleus^49^, while the autophagy is a cytoplasmic process. To be degraded in the vacuole, Sir2 needs to be transported from the nucleus into the cytoplasm. We thus examined the localization of Sir2 in WT and the H3T11A mutant expressing a Sir2-GFP fusion protein. Monitoring GFP by fluorescence microscopy showed that Sir2 was localized primarily in the nucleus in WT cells with only 25.08% cells having cytoplasmic-localized Sir2 (Fig. 5a, b). However, in H3T11A mutant, there was a significant increase (68.09%) of cells that have cytoplasmic-localized Sir2 (Fig. 5a, b), suggesting that H3T11A mutation accelerates the nuclear export of Sir2.

Analysis of Sir2 sequence revealed a consensus leucine (L)-rich nuclear export sequence (NES), LPEDLNSLYI, for the exportin chromosomal region maintenance 1 (Crm1). We thus examined Sir2 localization and protein levels in *TetO*_*7*_*-CRM1* mutant, where the *CRM1* promoter was replaced with TetO_7_ and *CRM1* transcription was shut off by doxycycline (Dox) treatment (Supplementary Fig. 6a)^34,50^. Knockdown of *CRM1* led to accumulation of Sir2 in the nucleus and impeded rapamycin-induced degradation of Sir2 (Fig. 5b–d and Supplementary Fig. 6a), indicating that the nuclear export of Sir2 is a prerequisite for its degradation by autophagy. By contrast, no consensus NES sequence was found in Sir3 and Sir4, which could explain why Sir2 but not Sir3 and Sir4 was degraded by autophagy.

We treated WT and H3T11A mutant with rapamycin and found that rapamycin treatment resulted in less Sir2 protein remaining in H3T11A compared to WT cells (Fig. 5e). To show that Sir2 degradation in H3T11A mutant is mediated by autophagy, WT and H3T11A mutant were treated with rapamycin along with PMSF or CQ. Rapamycin-induced Sir2 degradation in the H3T11A mutant can be restored by treatment with PMSF (Fig. 5f) and CQ (Fig. 5g). We also determined the effect of H3pT11 on Sir2 protein levels during chronological aging. H3pT11 levels were significantly reduced during chronological aging (Fig. 5h). The global levels of Sir2 were not only reduced during aging, but also significantly lower in H3T11A mutant when compared to WT (Fig. 5h). These data suggest that during chronological aging, H3T11 phosphorylation is reduced, which accelerates Sir2 degradation by autophagy.

We also deleted *PEP4*, which encodes a major vacuolar protease, in the H3T11A mutant. Loss of Pep4 restored Sir2 levels in the H3T11A mutant (Supplementary Fig. 6b). Similarly, the reduced Sir2 in *pyk1-ts* mutant was recovered in *pyk1-ts pep4Δ* mutant (Supplementary Fig. 6c). Moreover, the reduced Sir2 protein in the H3T11A mutant was increased to normal levels in H3T11A *atg2Δ* and H3T11A *atg12Δ* mutants (Fig. 5i and Supplementary Fig. 6d). The reduced Sir2 level was also recovered in *pyk1-ts atg2Δ* and *pyk1-ts atg12Δ* mutants (Fig. 5i and Supplementary Fig. 6e). Overall, these data indicate that SESAME-catalyzed H3pT11 prevents the nuclear export of Sir2 and inhibits autophagy-mediated Sir2 degradation.

### SESAME-catalyzed H3pT11 represses the transcription of autophagy genes and prevents the autophagic flux

**Fig. 6:**
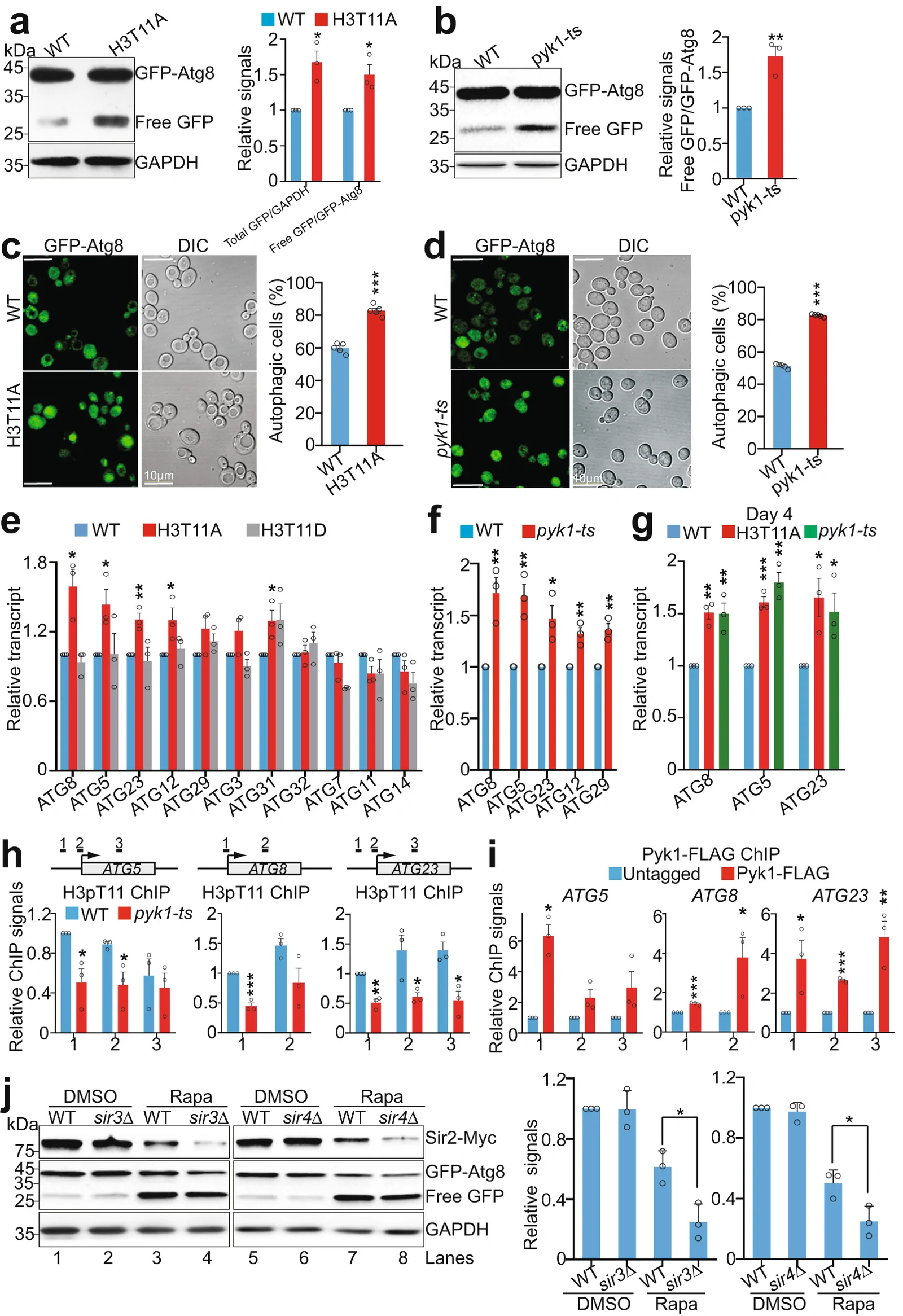
SESAME-catalyzed H3pT11 prevents the autophagy flux. **a** Representative immunoblot analysis of GFP-Atg8 and free GFP in WT and H3T11A mutant expressing the endogenous *ATG8* promoter-driven *GFP-ATG8* with anti-GFP antibody. GAPDH was used as a loading control. **b** GFP-Atg8 processing assays were performed in WT and *pyk1-ts* mutant expressing the endogenous *ATG8* promoter-driven *GFP-ATG8*. **c**, **d** Representative fluorescence microscopy images showed the distribution of GFP-Atg8 (green) in WT, H3T11A (**c**) and *pyk1-ts* (**d**) mutants. The autophagic cells were defined as cells with clear vacuolar GFP fluorescence. Quantification of autophagic cells depicted in Fig. 6c, d with 200–300 counts (blinded) for each replicate. **e**, **f** qRT-PCR analysis of the transcription of autophagy-related genes in WT, H3T11A (**e**), H3T11D (**e**), and *pyk1-ts* (**f**) mutants. **g** qRT-PCR analysis of the transcription of autophagy-related genes in WT, H3T11A, and *pyk1-ts* mutants when aged for 4 days in YPD medium. **h** Reduced H3pT11 enrichment at *ATG5*, *ATG8*, and *ATG23* in *pyk1-ts* mutant as determined by ChIP-qPCR using the amplicons at each gene as indicated at the top panel. **i** ChIP analysis of Pyk1 occupancy at *ATG5*, *ATG8*, and *ATG23*. **j** Loss of Sir3 and Sir4 accelerated rapamycin-induced Sir2 degradation. WT, *sir3Δ*, and *sir4Δ* mutants were treated with DMSO or rapamycin. The endogenously expressed Sir2 was detected with anti-Myc antibody. The autophagy activity was assessed by immunoblot analysis of GFP-Atg8 and free GFP. For Fig. 6a, b, e–j, the quantitative data represent means ± SE; *n* = 3 biological independent experiments. For Fig. 6c, d, the quantitative data represent means ± SE; *n* = 5 biological independent experiments. Statistical significance was tested using two-sided Student’s *t* test. *p < 0.05; **p < 0.01; ***p < 0.001.

To examine the effect of H3pT11 on autophagy, we employed a GFP (green fluorescent protein) liberation assay, which detects free GFP that is liberated upon the delivery of endogenous promoter-driven Atg8 with an N-terminal GFP tag (GFP-Atg8) to the vacuole and subsequent proteolysis of the Atg8 portion of the fusion protein^51^. The GFP liberation assay demonstrated significantly increased autophagic flux with active vacuolar proteolysis in H3T11A mutant compared to its WT counterpart as assessed by increased ratio of free GFP/GFP-Atg8 in H3T11A cells (Fig. 6a). Moreover, the amount of free GFP and GFP-Atg8 (total Atg8) was significantly higher in H3T11A mutant than that in WT, suggesting that H3pT11 could repress the expression of Atg8 (Fig. 6a). Consistently, there were much more free GFP proteins in H3T11A mutant than in WT during chronological aging (Supplementary Fig. 7a). We also noticed that the autophagy flux was significantly induced in *pyk1-ts* mutant (Fig. 6b). To strengthen these findings, we used a complementary assay by assessing the autophagy-dependent translocation of GFP-Atg8 to the vacuole by fluorescence microscopy. The percentage of autophagic cells that displayed clearly vacuolar localization of GFP, was significantly increased in H3T11A and *pyk1-ts* mutants (Fig. 6c, d). These data indicate that SESAME-catalyzed H3pT11 represses the autophagy pathway.

Previous studies showed that histone acetylation can modulate autophagy by regulating the expression of *ATG* genes^52^. Analysis of our ChIP-seq data showed that H3pT11 was enriched at *ATG* genes, especially *ATG31, ATG23, ATG8, ATG3,* and *ATG5* (Supplementary Figs. 1a, 7b). We therefore determined the relative mRNA abundance of *ATG* genes in WT and H3T11 mutants (H3T11A, H3T11D). The transcription of *ATG8*, *ATG5*, *ATG23*, *ATG12*, and *ATG31* was significantly increased in H3T11A but not in H3T11D mutants (Fig. 6e). Meanwhile, these genes were significantly induced in *pyk1-ts* mutant (Fig. 6f). Moreover, some of these *ATG* genes were significantly increased in H3T11A and *pyk1-ts* mutants when aged for 4 days (Fig. 6g). Thus, transcriptional control of the autophagy-relevant proteome may be crucial for enduring autophagic activity in H3T11A and SESAME mutants.

To test if SESAME-catalyzed H3pT11 was indeed causally linked to the transcriptional repression of *ATG* genes, we examined whether SESAME phosphorylates H3T11 at autophagy genes. As exemplified by *ATG5*, *ATG8*, *ATG23*, our ChIP-qPCR data confirmed that H3pT11 is enriched at these three genes (Fig. 6h). In *pyk1-ts* mutant, H3pT11 at these three genes was significantly reduced (Fig. 6h). We also detected significant binding of Pyk1 and Ser33 at these genes by ChIP-qPCR (Fig. 6i and Supplementary Fig. 7c), suggesting that SESAME phosphorylates H3T11 at *ATG* genes to repress their transcription and inhibit the autophagy flux.

Both Sir3 and Sir4 are required for Sir2 recruitment to telomeres and loss of Sir3 and Sir4 causes dissociation of Sir2 from telomeres^16^. We thus examined Sir2 protein levels in WT, *sir3Δ*, and *sir4Δ* mutants. Loss of Sir3 or Sir4 had no significant effect on autophagy as determined by the similar ratio of free GFP/GFP-Atg8 in WT, *sir3Δ*, and *sir4Δ* mutants (Fig. 6j, lanes 1 and 2, lanes 5 and 6). Accordingly, no significant difference was observed for Sir2 proteins in WT, *sir3Δ*, and *sir4Δ* mutants (Fig. 6j, lanes 1 and 2, lanes 5 and 6). However, when treated with rapamycin to induce autophagy, Sir2 was reduced to a significantly lower level in *sir3Δ* and *sir4Δ* mutants when compared with WT (Fig. 6j, lanes 3 and 4, lanes 7 and 8). Collectively, the above data suggest that both Sir2 dissociation and autophagy activation are required for efficient Sir2 degradation.

### SESAME-catalyzed H3pT11 regulates telomere silencing by promoting Sir2 binding at telomeres and preventing Sir2 degradation by autophagy

**Fig. 7:**
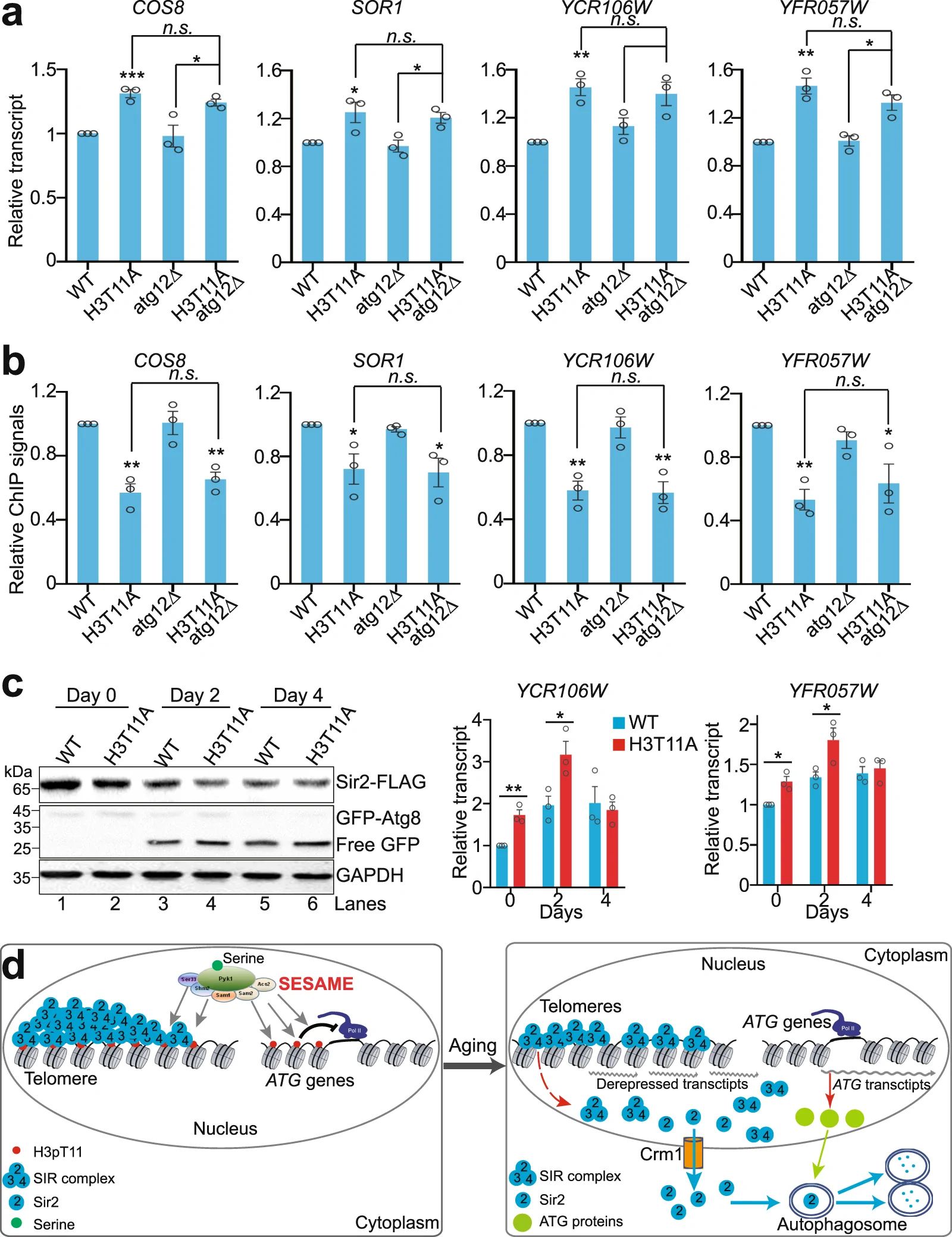
H3T11 phosphorylation maintains telomere silencing by promoting Sir2 binding at telomeres and preventing autophagy-mediated Sir2 degradation. **a** qRT-PCR analysis of *COS8*, *SOR1*, *YCR106W*, and *YFR057W* in WT, H3T11A, *atg12Δ*, and H3T11A *atg12Δ* mutants. **b** ChIP-PCR analysis of Sir2 occupancy at *COS8*, *SOR1*, *YCR106W*, and *YFR057W* in WT, H3T11A, *atg12Δ*, and H3T11A *atg12Δ* mutants. **c** Left panel: Western blots analysis of Sir2 and autophagy activity in WT and H3T11A mutant when aged for 0–4 days in YPD medium. Right panel: qRT-PCR analysis of *YCR106W* and *YFR057W* in WT and H3T11A mutant when aged for 0–4 days in YPD medium. For Fig. 7a–c, the quantitative data represent means ± SE; *n* = 3 biological independent experiments. Statistical significance was tested using two-sided Student’s *t* test. *p < 0.05; **p < 0.01; n.s. no significance. **d** Proposed model for SESAME-catalyzed H3pT11 maintains telomere silencing, promotes SIR complex binding at telomere regions, and prevents autophagy-mediated Sir2 degradation.

As H3pT11 prevents autophagy-mediated Sir2 degradation, we thus investigated whether blocking the autophagy pathway can restore the telomere silencing defects in H3T11A mutant. We examined the transcription of telomere-proximal genes in WT, H3T11A, *atg12Δ*, and H3T11A *atg12Δ* mutants. Although Sir2 protein levels were restored to normal levels in H3T11A *atg12Δ* mutant (Supplementary Fig. 6d), the transcription of telomere-proximal genes was significantly increased in both H3T11A and H3T11A *atg12Δ* mutants (Fig. 7a), indicating that blocking the autophagy pathway is not sufficient to rescue telomere silencing defects in H3T11A mutant. We then examined the occupancy of Sir2 at these genes in WT, H3T11A, *atg12Δ*, and H3T11A *atg12Δ* mutants. The Sir2 binding at these telomere-proximal genes was significantly reduced in both H3T11A and H3T11A *atg12Δ* mutants (Fig. 7b). These data suggest that H3T11 phosphorylation maintains telomere silencing both by promoting SIR complex binding at telomeres and preventing autophagy-mediated Sir2 degradation.

As Sir2 and H3pT11 levels were reduced during chronological aging, we thus examined the effect of H3pT11 on telomere silencing during chronological aging. WT and H3T11A mutant were aged for 0, 2, and 4 days in YPD media. During chronological aging, the autophagic activity was enhanced and the Sir2 protein levels were reduced in aging cells (Fig. 7c, lanes 3 and 5 vs lane 1). Accordingly, the transcription of telomere-proximal genes was significantly increased in both WT and H3T11A mutant (Fig. 7c), indicating a loss of telomere silencing during aging. Moreover, the Sir2 protein levels in H3T11A mutant was remarkably lower than those in WT when aged for 2 days (Fig. 7c, lane 3 vs lane 4). In parallel with Sir2 change, the transcription of these genes was significantly higher in H3T11A than that in WT when aged for 2 days (Fig. 7c). These data suggest that during chronological aging, the H3pT11 levels were reduced, which led to reduced Sir2 binding at telomeres, accelerated Sir2 degradation by autophagy, and compromised telomere silencing.

## Discussion

Telomere silencing in budding yeast is mediated by the assembly and spread of SIR complex. Heterochromatin assembly and transcription silencing are dynamically regulated by environmental stimuli, such as aging and cell metabolism^2,31,53^. How transcriptional silencing is dynamically regulated at telomeres remains largely unknown. Here, we show that the metabolic enzyme complex, SESAME regulates SIR complex occupancy at telomeres and transcriptional silencing in response to cell metabolism and chronological aging. Specifically, SESAME phosphorylates H3T11 at telomere regions, where it promotes the binding of SIR complex at telomeres and contributes to repression of subtelomeric genes. During chronological aging, H3pT11 is reduced, leading to dissociation and nuclear export of Sir2 to the cytoplasm in a Crm1-dependent manner. The cytoplasmic Sir2 is degraded by autophagy. Meanwhile, loss of H3pT11 upregulates the transcription of autophagy-related genes, i.e., *ATG5*, *ATG8*, and *ATG23* to increase the autophagy flux. As a consequence, Sir2 is degraded by autophagy machinery and telomere silencing is compromised. Moreover, serine stimulates SESAME to phosphorylate H3T11 to increase Sir2 and enhance telomere silencing. Thus, our study reveals a function of SESAME-catalyzed H3pT11 in maintenance of Sir2 homeostasis and telomere silencing in response to cell metabolism and aging (Fig. 7d).

Histone modifications play important roles in regulation of telomere silencing by restricting the spread of SIR complex into euchromatic regions in budding yeast, including Sas2-catalyzed H4K16ac^54^, Dot1-catalzyed H3K79me3^20^, and Set1-catalyzed H3K4me3^21^. These modifications occur predominantly in euchromatin regions and thus affect heterochromatin in an indirect manner^22^. The silent heterochromatin contains histone H2AS129 phosphorylation, H3R2 asymmetrical demethylation (H3R2me2a), H4K20 monomethylation (H4K20me1) and H4K12ac^12,55–58^. However, their functions at telomeres have not been explored extensively. Here, we identified H3pT11 as a histone marker that is enriched in telomere regions and promotes SIR complex occupancy at telomeres. The function of SESAME and H3pT11 at telomere heterochromatin regions is independent on Set1-catalyzed H3K4me3. As SESAME-catalyzed H3pT11 regulates telomere silencing but has no effect on silencing at mating-type loci, it is possible that H3pT11 could indirectly regulate telomere silencing. Our ChIP-seq data also showed that at some silent loci, the localization of H3pT11 is immediately adjacent to SIR binding sites, suggesting that H3pT11 may function as a boundary like other histone modifications, i.e., H3K4me3 and H3K79me3 to block the spread of SIR complex. Moreover, H3pT11 is highly enriched at the transcription start site (TSS) of highly transcribed genes and Gcn5 has a preference towards H3T11 phosphorylation^59^. It is likely that H3T11A mutant strongly affects highly active promoters across the genome, which then indirectly lead to shifting of SIR complex to other sites across the genome and altered telomere structure. In addition, some factors have been reported to indirectly regulate the binding of active histone modifiers at heterochromatin. For example, loss of H3K79 methylase Dot1 leads to rearrangement of heterochromatin and one plausible mechanism is promoting the binding of histone acetyltransferase Gcn5 at heterochromatin^60^. Moreover, MRG1, which a factor exclusively bound at euchromatin in *Caenorhabditis elegans*, promotes the anchoring of heterochromatin at the nuclear periphery^61^. Loss of MRG1 increases the occupancy of histone acetyltransferase CBP-1 and H3K27ac at heterochromatin^61^. Hence, it is possible that SESAME and H3pT11 may prevent the mislocalization of euchromatic factors and/or histone modifications at telomere heterochromatin.

Telomere silencing is regulated by cell metabolism. Although the relationship between glucose deficiency, NAD^+^, and telomere silencing is complex, recent studies emphasize the importance of examining the nuclear functions of metabolic enzymes in modulating telomere silencing. For example, the glyceraldehyde 3-phosphate dehydrogenase (Tdh3) and glutamate dehydrogenase 1 (Gdh1) regulate the activity of Sir2 and telomere silencing by affecting the nuclear NAD^+^ and α-ketoglutarate levels, respectively^31,32^. In addition, Gas1, a β-1,3-glucanosyltransferase, has been shown to regulate telomere silencing by interacting with Sir2 and perhaps adding β-1,3-glucan to Sir2^62^. Unlike these enzymes, pyruvate kinase-containing SESAME complex epigenetically regulates telomere silencing independent of NAD^+^ and α-ketoglutarate. SESAME directly binds to telomeres, phosphorylates H3T11, and maintains telomere silencing by promoting SIR complex occupancy and preventing autophagy-mediated Sir2 degradation. The homolog of pyruvate kinase in mammals, PKM2 has been shown as a telomere-binding protein by tandem affinity purification coupled with the label-free comparative mass spectrometry despite little being known about its effect on telomere structure^63^. Given the high similarity between PKM2 and Pyk1, it is possible that PKM2 may regulate telomere silencing in mammals like Pyk1. Moreover, we found a function of serine and serine metabolic enzymes (Ser33 and Shm2) in regulating telomere silencing by stimulating Pyk1-catalyzed H3pT11 and preventing Sir2 degradation. Hence, serine metabolism enhances telomere silencing by inducing SESAME-catalyzed H3pT11 and maintaining Sir2 homeostasis.

During carbon source exhaustion in the aging process, the long-lived quiescent cells group their telomeres into a unique focus or hypercluster localized in the center of the nucleus^24^. The telomere hyperclusters correlate with more stable telomere silencing^25^. Although we observed that H3pT11 prevents the nuclear export of Sir2 during the aging process (Extended Data Fig. 8a), H3pT11 has no clear effect on telomere hyperclustering (Extended Data Fig. 8b). These data suggest that H3pT11 does not maintain telomere silencing by promoting the formation of telomere hyperclusters. This could be explained by the fact that telomere hyperclustering in quiescent cells is not driven by an increase of Sir3 spreading and gene silencing is not necessary for telomere hyperclustering^24,25^.

Autophagy is an evolutionarily conserved cellular process that primarily participates in lysosome-mediated protein degradation. It has been reported that SIRT1, the Sir2 homolog in mammals, promotes autophagy^64^; however, little is known about the effect of autophagy on SIRT1 (Sir2) protein level. We find that Sir2 protein stability is controlled by autophagy, which interestingly is regulated by H3pT11. Our findings thus establish autophagy as an important regulator of telomere silencing. Notably, autophagy is responsible for the loss of Sir2 and transcriptional silencing during chronological aging. By focusing on SESAME and H3pT11, we not only link autophagy and telomere silencing but also show how they are dynamically controlled: during the aging process, H3pT11 is reduced, which leads to reduced Sir2 binding and accelerated nuclear export of Sir2; meanwhile, reduced H3pT11 induces autophagy to degrade Sir2. By analyzing the global genetic interaction network generated by Costanzo et al.^65^, we find a negative genetic interaction between *PYK1* and autophagy-related genes (*ATG3*, *ATG4*), consistent with our findings that Pyk1 represses autophagy. Knockdown of pyruvate kinase PKM2 has been shown to induce autophagy in mammalian cells^66^. As PKM2 has also been shown to phosphorylate H3T11^67^, it is possible that PKM2-phosphorylated H3T11 may prevent autophagy-degradation of Sir2 homologs in mammals. In agreement with the role of Pyk1 and H3pT11 in autophagy, the SESAME subunit, Acs2 has also been reported to repress autophagy-related gene transcription^52^, suggesting the concerted function of SESAME subunits in regulation of autophagy.

We identified a histone modification that prevents autophagy flux by repressing *ATG* gene expression. Autophagy has been reported to be regulated by few histone modifications. Under starvation conditions, the reduced H2B monoubiquitination (H2Bub1) results in the activation of autophagy by controlling the transcription of autophagy regulatory genes^68^. Rpd3-mediated histone deacetylation represses autophagy^69^. The acetyltransferase hMOF catalyzed H4K16ac is involved in autophagy regulation^70^. Here, we show that H3pT11 inhibits autophagy by directly repressing the transcription of autophagy genes. It remains unclear how H3pT11 represses the transcription of autophagy genes.

Together, our study reveals a pathway by which metabolism can influence SIR-mediated silencing through the SESAME complex. We identify SESAME-catalyzed H3pT11 as an epigenetic regulator of telomere heterochromatin structure. Our results also reveal functions of autophagy in regulating telomere silencing.

## Methods

### Materials

All yeast strains used in this study are described in Supplementary Table 1. The gene deletion mutants and genomic integration of C-terminal tags were constructed by homologous recombination of PCR fragments^71^. Fusion of the tags (FLAG, Myc) to individual SESAME subunits and Sir2 does not interfere with the functions of the proteins studied as no defects in cell growth, histone modifications, and telomere silencing were observed. All yeast strains were verified by colony PCR, DNA sequencing, qRT-PCR, and/or Western blots before used for experiments. The sequence of primers used for qPCR is listed in Supplementary Table 2.

### Constructs and cloning

The plasmid that overexpresses Sir2 (*pTEFpro-SIR2*) was constructed as follows: *SIR2* was amplified by primers (Forward: 5′-TTTCTAGAACTAGTGGATCCATGACCATCCCACATATGAAA-3′; Reverse primer 5′-AGAAAACGTGAAACAAGCCCCAAATATGCATGTCTGGTTAACTATAGGGCGAATTGGGT-3′ in Supplementary Table 2 using yeast genomic DNA as the template. The amplified *SIR2* fragment was digested with T5 exonuclease and then ligated into the *pTEF* vector using the seamless cloning followed by verification by DNA sequencing.

### Cell growth and treatment

The concentration of rapamycin, PMSF, CQ, and 3MA used is 1 μg/ml, 5 mM, 10 mM, and 50 μM, respectively. For Fig. 4a, cells were grown in YPD medium to OD_600_ of 0.5 and treated with 5 mM MG132 or 5 mM PMSF for 2 h. For Fig. 4b–d, cells were treated with 50 μM 3-MA, 5 mM PMSF, 10 mM CQ, and 5 mM MG132 along with 1 μg/ml rapamycin for 2 h. For Fig. 4e, f, cells were treated with 1 μg/ml rapamycin for 0–1.5 h. For Supplementary Fig. 5a–d, cells were treated with 1 μg/ml rapamycin for 1 h. For Fig. 5c, d, cells were treated with 6.25 μg/ml doxycycline for 1.5 h.

### Immunoblot analysis

Proteins were extracted from exponentially growing yeast cells^33,72^. Cells were grown in 5 ml YPD (Yeast Extract Peptone Dextrose) or selective medium as indicated until OD_600_ of 0.7–1.0. Cells were harvested and lysed in alkaline lysis buffer (2 M NaOH, 8% (v/v) β-mercaptoethanol) on ice for 15 min. After centrifugation, the protein pellet was resuspended in 150 μl 2×SDS-sample buffer. Protein samples were separated by 8–15% SDS-PAGE and transferred to Immobilon-P PVDF membrane. The blots were probed with primary antibodies followed by incubation with horseradish peroxidase-labeled IgG secondary antibodies. The protein bands were visualized using the ECL Chemiluminescence Detection Kit (Bio-Rad, 170–5061) and quantified with Image J software (v.1.8.0) (https://imagej.en.softonic.com/). All Western blots were performed at least three times and one typical example was shown.

### Chromatin immunoprecipitation (ChIP) assay

Yeast cells were grown in 200 ml YPD media at 30 °C until OD_600_ of ~0.7–1.0. The crosslinking was performed in 1% formaldehyde and quenched by adding 10 ml of 2.5 M glycine. Harvested cells were washed once with TBS + PMSF pre-chilled to 4 °C, lysed with glass beads in FA-SDS lysis buffer (0.1% SDS, 40 mM HEPES-KOH, pH7.5, 1 mM EDTA pH 8.0, 1% Triton X-100, 0.1% Na deoxycholate, 1 mM PMSF, 2 μg/ml leupeptin, 1 μg/ml pepstatin A, protease inhibitor cocktail, phosphatase inhibitor cocktail). DNA was sonicated to an average size of 500 bp and subjected to immunoprecipitation with anti-H3 (2 μl; ab1791, Abcam), anti-H3pT11 (3 μl; ab5168, Abcam), anti-FLAG antibody (5 μl, F1804, Sigma), anti-H4K16ac (2 μl) pre-bound to Protein G Dynabeads (Invitrogen) at 4 °C overnight. The beads were subsequently washed with FA lysis buffer, FA buffer + 1 M NaCl, FA buffer + 0.5 M NaCl, TEL buffer (10 mM Tris pH 8.0, 1 mM EDTA, 0.25 M LiCl, 1% NP-40, 1% Na deoxycholate), and TE (10 mM Tris pH 7.4, 1 mM EDTA). The eluted DNA/protein complexes were treated with 20 µg Proteinase K at 55 °C for 1 h and then treated at 65 °C overnight. The DNA was digested by RNase (Roche), purified with ethanol precipitation and quantitated by qPCR with primers listed in Supplementary Table 2. For ChIP-qPCR in Figs. 2h–j, 6h, i and 7b, Supplementary Figs. 1c, d, 3c–h, 7c, the percentage IP was calculated and values were then plotted with wild-type or untagged cells normalized to 1.

ChIP-seq was done for four biological replicates with anti-H3pT11 and anti-H3 antibodies^33^. Library was constructed and sequenced on an Illumina platform^33^. Data was aligned to yeast genome sacCer3 from UCSC using bowtie2 version 2.1.0 with parameter -k 1. As the telomere repeated sequences cannot be mapped to specific telomeres, they were excluded for further analysis. Data was read into R (3.1.0) for further analysis. Peaks were called using MACS2 (v.2.1.1, macs2 callpeak) with parameter -t -c -g 1.2e7 -n -B -q 0.01–nomodel. Peaks annotation was performed on a website service (https://manticore.niehs.nih.gov/pavis2/). Tracks was smoothed by deepTools2 (v.2.0) and visualized by IGV software (v.2.0) with a reference genome of *S. cerevisiae* (sacCer3). The control tracks were generated using untagged BY4741 strain with anti-FLAG antibody. The raw ChIP-seq dataset for Sam1 and Pyk1 is derived from GSE72972. The raw ChIP-seq dataset for Sir2, Sir3, and Sir4 is derived from SRP030670. Raw data were downloaded by SRA toolkit (v.2.9.2). Files were unzipped and filtrated by Fastqc (v.0.11.9) and Trim Galore (v.2.11).

### Quantitative reverse transcription PCR (qRT-PCR)

Total RNA was isolated from exponentially growing yeast cells by standard phenol-chloroform extraction procedures. The purified RNA was digested with DNase I (RNase-free) (Takara catalog no. 2270A) at room temperature for 30 min and quantified by Nanodrop 2000 (Thermo scientific). The integrity of RNA was assessed by agarose gel electrophoresis. 500 ng total RNA was used for reverse transcription PCR (RT-PCR) in a 10 μl reaction volume with Reverse Transcriptase Kit (M-MLV) (ZOMANBIO)^33,72^. qPCR was carried out on a Bio-Rad real-time PCR machine with iTaq™ Universal SYBR^®^ Green Supermix (Bio-Rad, Cat: 1725121). Primers used for qRT-PCR are described in Supplementary Table 2. We used 2^(-ΔΔCt)^ to determine the quantity of relative transcription level. The mRNA level of the gene of interest was normalized to that of beta-actin.

### Microscopy analysis

Yeast cells were cultured in YPD or selective medium until OD_600_ of 1.0. After washing with cold phosphate buffered saline once, the cells were resuspended in phosphate buffered saline. Cells were fixed with 4% formaldehyde in phosphate buffered saline for 30 min and treated with DAPI for 15 min at room temperature. Cells were then washed with cold phosphate buffered saline. The cell morphology was visualized using a ZEISS LSM710 microscope (Germany) with a 100× oil immersion objective by fluorescent microscopy. Images were acquired using ZEN Imaging Software ZEN 2.1 (ZEISS). DAPI was used to indicate the nucleus. The merged color Images were generated by Image J.

### Antibodies

All antibodies used in this study were listed in Supplementary Table 3. Antibodies against anti-H3 (1: 5000; ab1791) and H3T11 phosphorylation (1:5000; ab5168) were purchased from Abcam; antibodies against Sir2 (1:500; sc-6667), and Sir3 (1:500; sc-101612) were purchased from Santa Cruz Biotechnology; antibodies against GAPDH (1:10000; 10494-1-AP), GFP (1:5000; 66002-1-1g), Myc (1:5000; 60003-2-1g), goat polyclonal anti-mouse IgG (1:5000; SA00001-1), and goat polyclonal anti-rabbit IgG (1:5000; SA00001-2) were obtained from proteintech; antibody against histone H3 (1:3000; 9715S) was purchased from Cell Signaling Technology; antibody against FLAG M2 (1:3000; F1804-1MG) was obtained from Sigma-Aldrich; antibody against H4K16ac (1:2000; 07-329) was obtained from EMD Millipore.

### Statistics and reproducibility

Representative results of at least three biological independent experiments were performed in all of the figure panels. The two-sided Student’s *t* test in Microsoft Excel (professional Plus2013) was used for comparison between two groups and a *p*-value <0.05 was considered statistically significant. *p < 0.05; **p < 0.01; ***p < 0.001; n.s. no significance. For all error bars, data are mean ± standard error (SE).

### Reporting summary

Further information on research design is available in the Nature Research Reporting Summary linked to this article.

## Supplementary information

Supplementary Information

Reporting summary

## Source data

Source Data

## Data Availability

The accession code for ChIP-seq of H3 and H3pT11 is GSE147050. The accession code for ChIP-seq of control, Ser33, and Pyk1 is GSE72972. The accession code for ChIP-seq of H3 and H3K4me3 is GSE115910. The accession code for ChIP-seq of Sir2, Sir3, and Sir4 is SRP030670. All data supporting the findings of this study are included in the manuscript and its supplementary files or are available from the corresponding author upon request. Source data for the figures and supplementary figures are provided as a Source Data file.
