# Author Correction: Metabolic regulation of telomere silencing by SESAME complex-catalyzed H3T11 phosphorylation

**DOI:** 10.1038/s41467-026-78172-x

**Published:** 2026-10-29

**Authors:** Shihao Zhang, Xilan Yu, Yuan Zhang, Xiangyan Xue, Qi Yu, Zitong Zha, Madelaine Gogol, Jerry L. Workman, Shanshan Li

**Affiliations:** 1 State Key Laboratory of Biocatalysis and Enzyme Engineering, College of Life Sciences, Hubei University, Wuhan, Hubei 430062 China; 2 Human Aging Research Institute (HARI), School of Life Science, Nanchang University, Nanchang, Jiangxi 330031 China; 3 Stowers Institute for Medical Research, 1000 E. 50th Street, Kansas City, MO 64110 USA

Correction to: *Nature Communications* https://doi.org/10.1038/s41467-020-20711-1, published online 26 January 2021

In the version of this article initially published, due to data preparation mistakes, there were errors in figures and associated Source Data affecting the quantitation bar graph for *TEL-12L* (*TEL XII L*) and *PYK1* in Fig. 1f; the quantitation bar graph for *ATG8* (*H3T11A*) in Fig. 6g; the quantitation bar graph for *SOR1* (*sam1Δ*) in Fig. S2c, and the quantitation bar graph for *SOR1* (glycine) in Fig. S2f. The figures and Source Data are now updated in the HTML and PDF versions of the article. For comparison, original figures are available as Supplementary Information accompanying this amendment.

Incorrect Figure 1f 

Correct Figure 1f 

Incorrect Figure 6g

Correct Figure 6g

