# Supplementary material for: Metabolic regulation of telomere silencing by SESAME complex-catalyzed H3T11 phosphorylation
Source: Nat Commun. 2021 Jan 26;12:594. doi: 10.1038/s41467-020-20711-1 (PMC7838282; doi:10.1038/s41467-020-20711-1)
Supplement: Supplementary file 1 — Supplementary Information [file 41467_2020_20711_MOESM1_ESM.pdf]

Supplementary Information

Metabolic regulation of telomere silencing by SESAME complex-catalyzed H3T11 phosphorylation

Shihao Zhang[1,4], Xilan Yu[1,4], Yuan Zhang[1], Xiangyan Xue[1], Qi Yu[1], Zitong Zha[2], Madelaine Gogol[3], Jerry L. Workman[3], Shanshan Li[1,*]

[1]State Key Laboratory of Biocatalysis and Enzyme Engineering, College of Life Sciences, Hubei University, Wuhan, Hubei 430062, China

[2]Human Aging Research Institute (HARI), School of Life Science, Nanchang University, Nanchang, Jiangxi 330031, China

[3]Stowers Institute for Medical Research, 1000 E. 50th Street, Kansas City, MO 64110, USA

[4]These authors contribute equally to this work

*Corresponding author

Email: shl@hubu.edu.cn

Supplementary Fig. 1

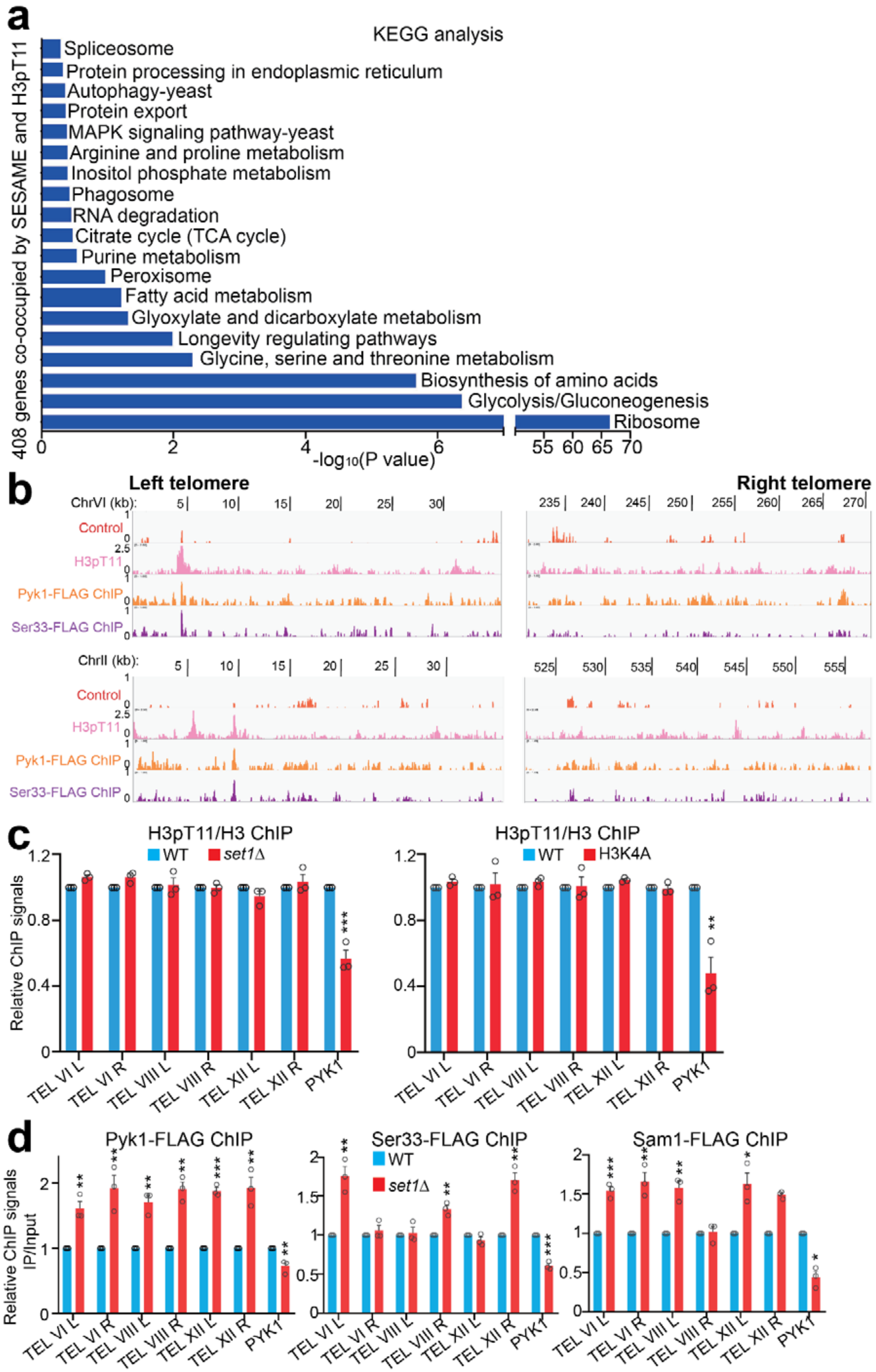

Supplementary Fig. 1 SESAME phosphorylates H3T11 at telomere regions.

a, KEGG analysis of 408 genes co-occupied by the SESAME complex and H3pT11.

b, ChIP-seq tracks showing the occupancy of H3pT11, Pyk1 and Ser33 at the left and right telomere regions on chromosome VI (TEL VI) and chromosome II (TEL II).

c, Set1 and H3K4me3 had no significant effect on H3pT11 enrichment at telomeres. ChIP-qPCR analysis of H3pT11 enrichment at chromosome VI (TEL VIL and TEL VIR), chromosome VIII (TEL VIIIL and TEL VIIIR) and chromosome XII (TEL XIIL and TEL XIIR) in WT, *set1Δ* and H3K4A mutants. The *PYK1* gene was used as a positive control.

d, ChIP-qPCR analysis of Pyk1, Ser33 and Sam1 at telomeres of chromosome VI (TEL VIL and TEL VIR), chromosome VIII (TEL VIIIL and TEL VIIIR) and chromosome XII (TEL XIIL and TEL XIIR) in WT and *set1Δ* mutant. Although the occupancy of Pyk1, Ser33 and Sam1 was significantly reduced at *PYK1* region, the enrichment of SESAME at telomeres was not reduced but slightly increased, suggesting that Set1 is not required for SESAME binding at telomeres.

For Supplementary Fig. 1c and 1d, the quantitative data represent the mean ± SE; n=3 biological independent experiments. Statistical significance was tested using two-sided Student’s t-test. *, $p<0.05$; **, $p<0.01$; ***, $p<0.001$.

Supplementary Fig. 2

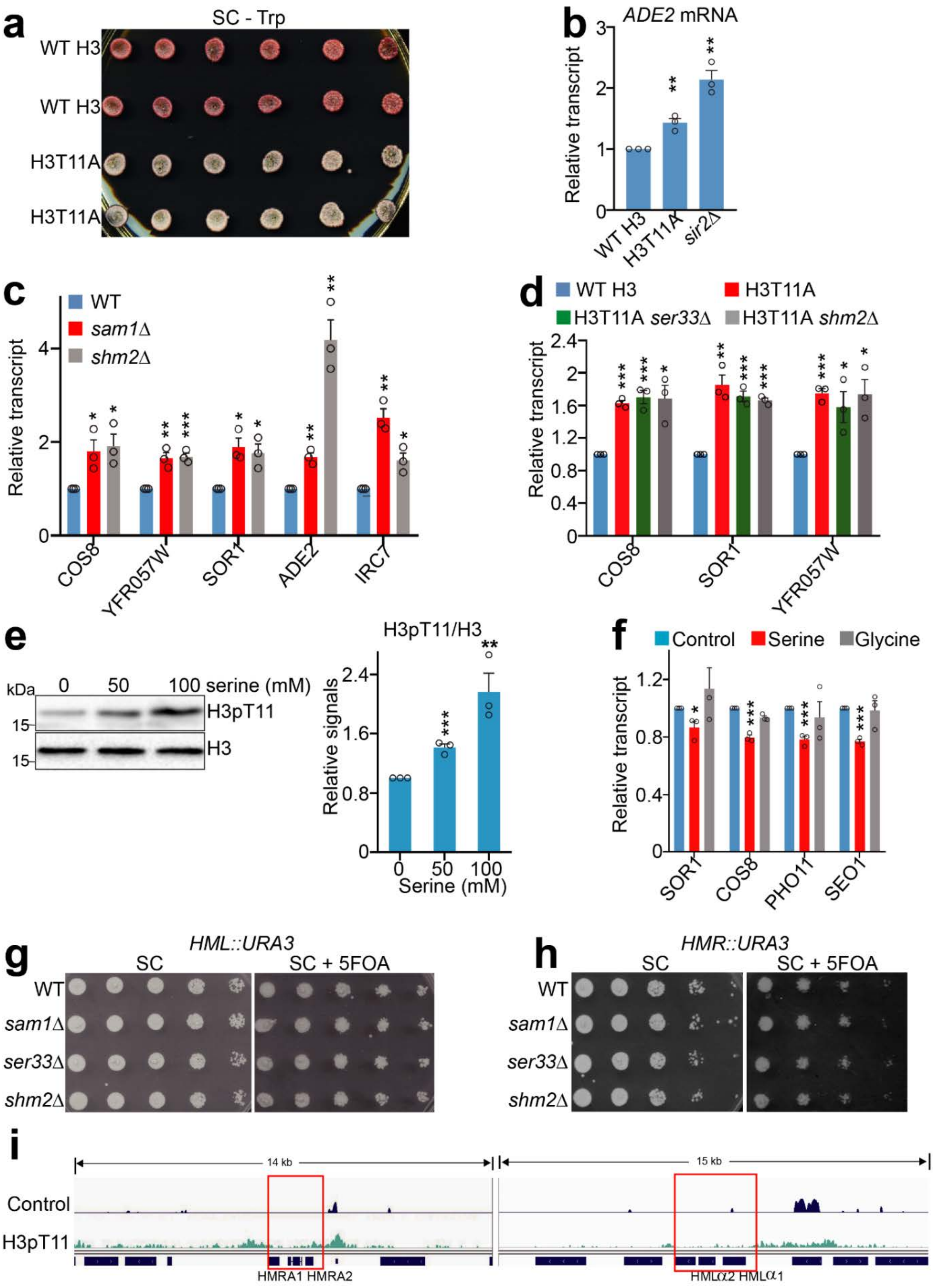

Supplementary Fig. 2 SESAME-catalyzed H3T11 phosphorylation maintains normal telomere silencing.

a, H3pT11 is required to maintain telomere silencing. WT and H3T11A mutant bearing *ADE2* adjacent to telomere repeats on chromosome V-R (Tel V-R) were grown to saturation, normalized for OD_{600}, 3-fold serially diluted and spotted on SC-Trp plate and grown at 28 ℃ for 4-6 days. The red color of spots indicates intact *ADE2* silencing whereas the white color indicates diminished silencing of *ADE2*. The images shown are a typical example of three independent experiments.

b, qRT-PCR analysis of *ADE2* transcription in WT, H3T11A and *sir2Δ* mutants.

c, qRT-PCR analysis of the transcription of *COS8*, *YFR057W*, *SOR1*, *ADE2*, and *IRC7* in WT, *sam1Δ* and *shm2Δ* mutants. *IRC7* localizes 4.9 kb from telomere VI-R.

d, qRT-PCR analysis of the transcription of *COS8*, *SOR1* and *YFR057W* in WT, H3T11A, H3T11A *ser33Δ* and H3T11A *shm2Δ* mutants.

e, Serine significantly increased the global H3pT11 as determined by Western blots.

f, qRT-PCR analysis of the effect of serine and glycine on the transcription of *SOR1*, *COS8*, *PHO11* and *SEO1*.

g and **h**, WT, *sam1Δ*, *ser33Δ*, and *shm*2Δ cells bearing *URA3* adjacent to *HML* (**g**) or *HMR* (**h**) were grown to saturation, normalized for OD_{600}, 3-fold serially diluted and spotted on synthetic complete (SC) and SC + 5-FOA plates. Cell growth was monitored at 28 ℃. The images shown are a typical example of three independent experiments.

i, ChIP-seq tracks showing the occupancy of H3pT11 at representative *HML* and *HMR* loci.

For Supplementary Fig. 2b, 2c, 2d, 2e and 2f, the quantitative data represent means ± SE; n=3 biological independent experiments. Statistical significance was tested using two-sided Student's t-test. *, $p<0.05$; **, $p<0.01$; ***, $p<0.001$.

Supplementary Fig. 3

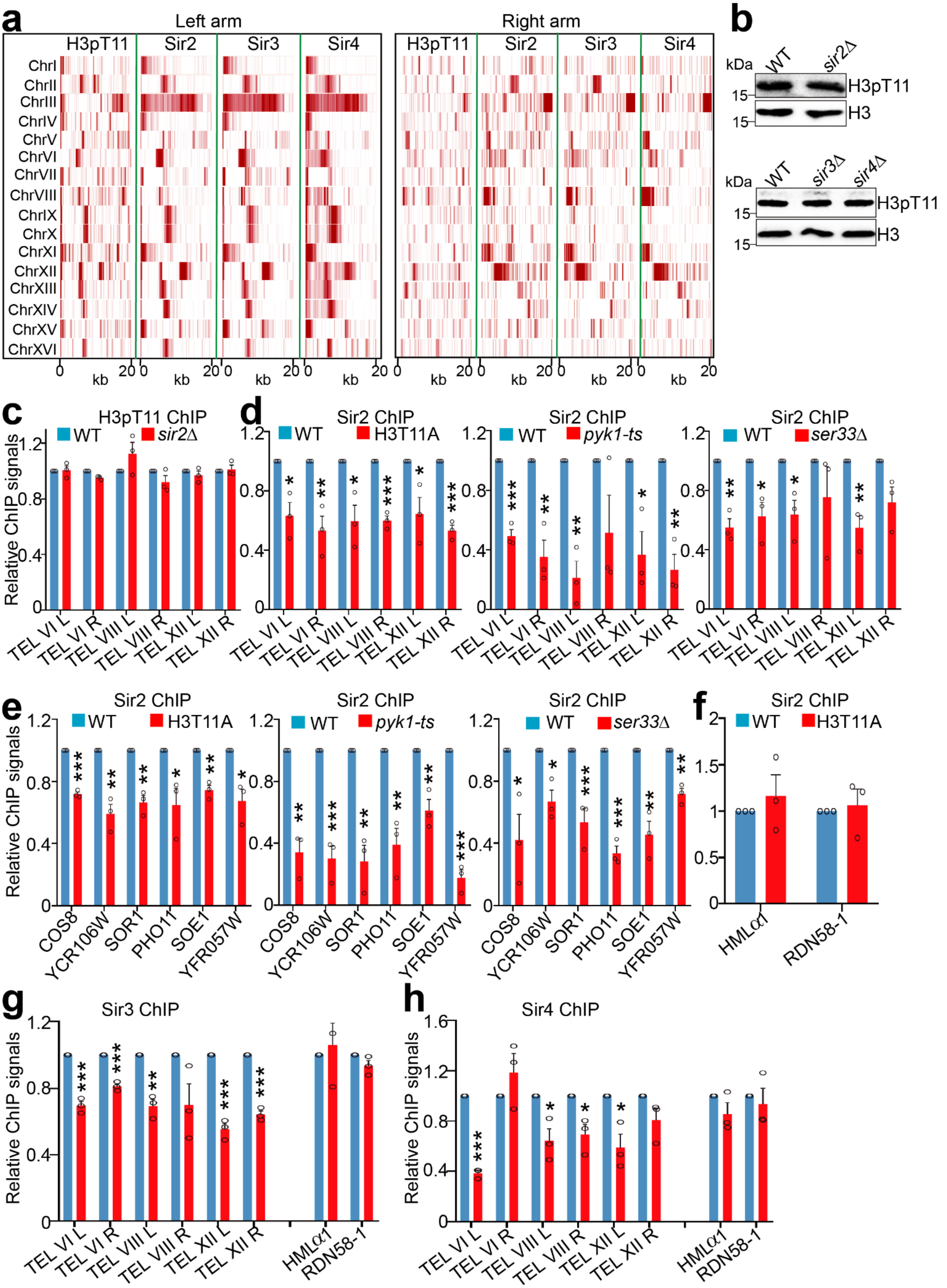

Supplementary Fig. 3 SESAME-catalyzed H3T11 phosphorylation promotes SIR complex binding at telomere regions.

a, Heatmap showing the occupancy of H3pT11 and SIR (Sir2/Sir3/Sir4) complex at regions within 20 kb of telomeres in left and right arms of all chromosomes.

b, Western blots analysis of H3pT11 in WT, *sir2Δ*, *sir3Δ* and *sir4Δ* mutants.

c, ChIP-qPCR analysis of H3pT11 at telomeres of chromosome VI (TEL VIL and TEL VIR), chromosome VIII (TEL VIIIL and TEL VIIIR) and chromosome XII (TEL XIIL and TEL XIIR) in WT and *sir2Δ* mutant.

d, ChIP analysis of Sir2 occupancy at telomere VI (TEL VIL and TEL VIR), VIII (TEL VIIIL and TEL VIIIR) and XII (TEL XIIL and TEL XIIR) in WT, H3T11A, *pyk1-ts* and *ser33Δ* mutants.

e, ChIP analysis of Sir2 occupancy at telomere proximity genes in WT, H3T11A, *pyk1-ts* and *ser33Δ* mutants.

f, ChIP analysis of Sir2 occupancy at *HMLα1* and *RDN58-1* in WT and H3T11A mutant.

g and **h**, ChIP analysis of Sir3 and Sir4 occupancy at telomeres of chromosome VI (TEL VIL and TEL VIR), chromosome VIII (TEL VIIIL and TEL VIIIR), chromosome XII (TEL XIIL and TEL XIIR), *HMLα1* and *RDN58-1* in WT and H3T11A mutants.

For Supplementary Fig. 3b, shown are the typical example of three biological independent replicates. For Supplementary Fig. 3c-3h, the quantitative data represent means ± SE; n=3 biological independent experiments. Statistical significance was tested using two-sided Student’s t-test. *, $p<0.05$; **, $p<0.01$; ***, $p<0.001$.

Supplementary Fig. 4

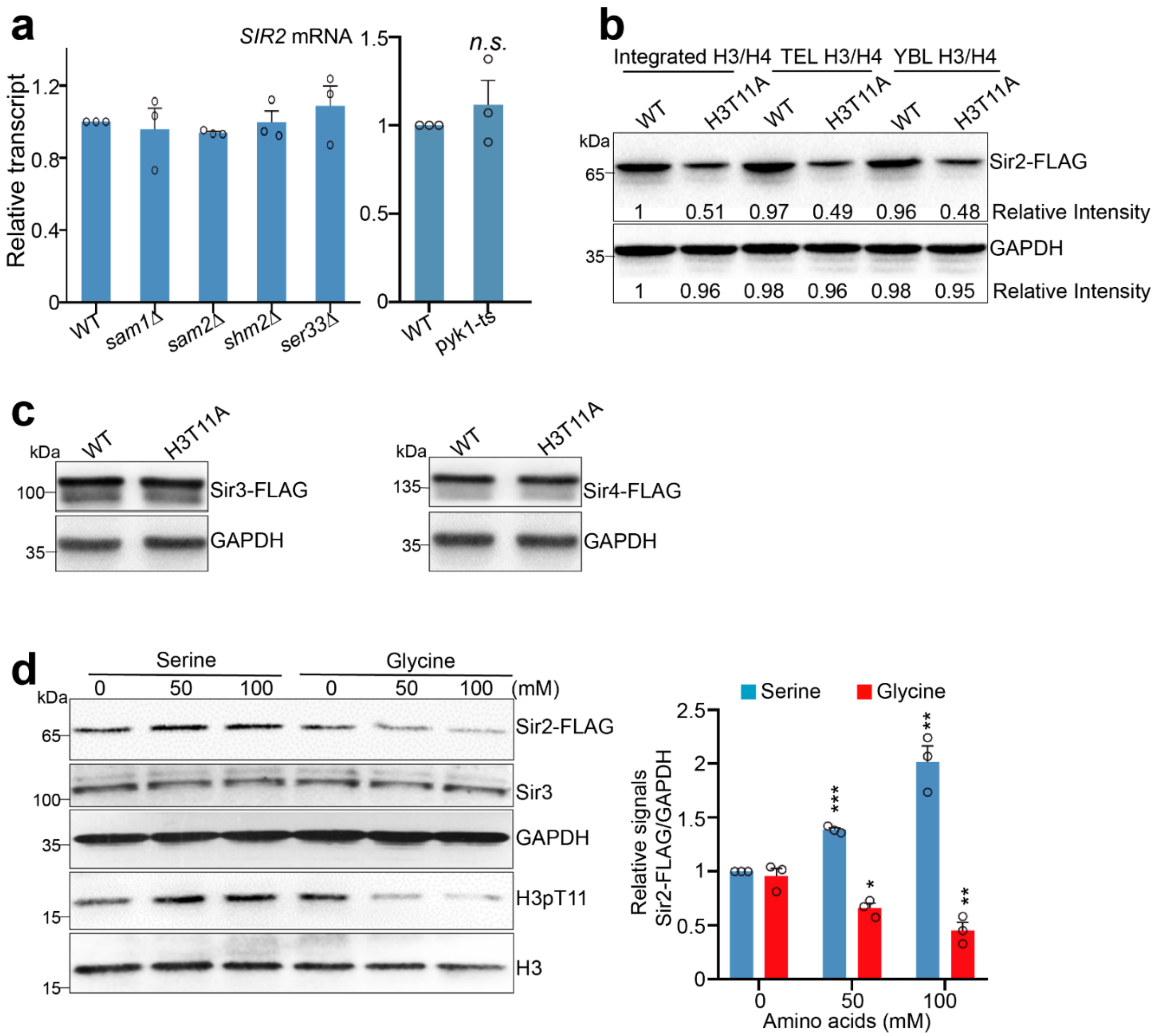

Supplementary Fig. 4 SESAME-catalyzed H3T11 phosphorylation is required to maintain normal Sir2 protein levels.

a, qRT-PCR analysis of *SIR2* expression in WT, *sam1Δ*, *sam2Δ*, *shm2Δ*, *ser33Δ* and *pyk1-ts* mutants.

b, Western blots analysis of Sir2 and GAPDH in different genetic backgrounds of WT and H3T11A mutants. All strains used contain only one copy of H3 and H4 genes. The integrated H3/H4 strains contain H3 and H4 in the genome. The TEL H3/H4 (UCC1369) and YBL H3/H4 (YBL574) strains harbor a *HHF1-HHT1* containing plasmid that encodes H3 and H4. TEL H3/H4 (H3T11A) and YBL H3/H4 (H3T11A) mutants were constructed by transforming the plasmids containing H3T11A mutation into UCC1369 and YBL574 and selecting on α-aminoadipic acid or 5-FOA plates to remove the wild-type histone plasmid as described [1].

c, Western blots analysis of Sir3 and Sir4 in WT and H3T11A mutant. H3T11A had no significant effect on Sir3 and Sir4 protein levels.

d, Serine but not glycine treatment significantly increased the overall Sir2 protein levels. Cells were treated with different concentrations of serine or glycine. The endogenous Sir2-FLAG and Sir3 were detected with anti-FLAG and anti-Sir3 antibodies, respectively. The H3pT11 was examined with anti-H3pT11 antibody.

For Supplementary Fig. 4b and 4c, shown are the typical example of three biological independent replicates.

For Supplementary Fig. 4a and 4d, the quantitative data represent means ± SE; n=3 biological independent experiments. Statistical significance was tested using two-sided Student’s t-test. *, $p<0.05$; **, $p<0.01$; ***, $p<0.001$.

Supplementary Fig. 5

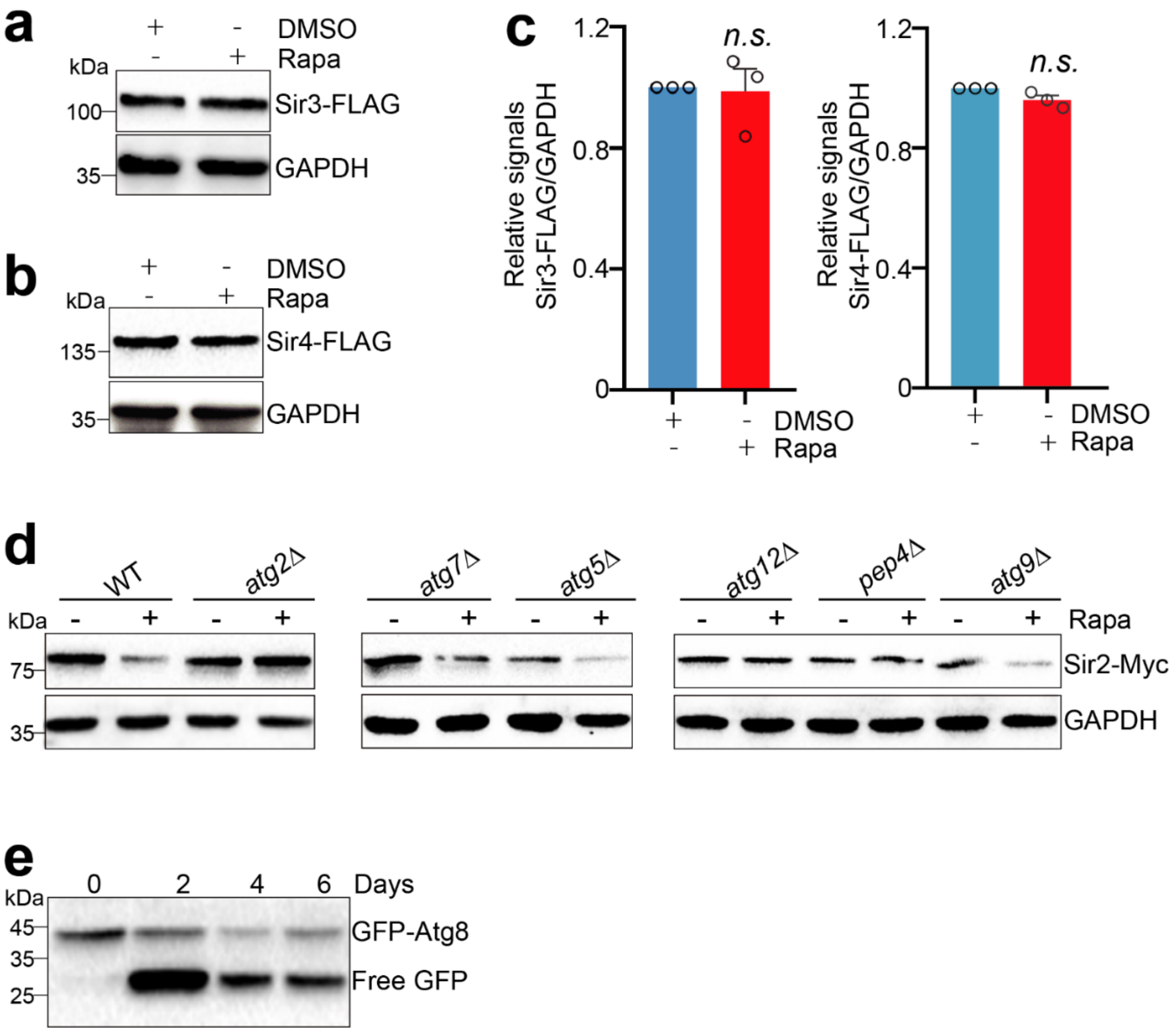

Supplementary Fig. 5 Sir2 can be degraded by autophagy.
a-c, Rapamycin had no significant effect on Sir3 and Sir4 protein levels. Cells were treated with DMSO or 1 μg/ml rapamycin for 1 hr and the global levels of endogenously expressed Sir3-FLAG and Sir4-FLAG were detected with anti-FLAG antibody.
d, Western blots analysis of Sir2 protein levels in WT, *atg2Δ*, *atg5Δ*, *atg7Δ*, *atg9Δ*, *atg12Δ* and *pep4Δ* mutants when treated with DMSO or 1 μg/ml rapamycin (Rapa) for 1 hr.
e, Autophagy was induced during chronological aging as determined by a GFP (green fluorescent protein) liberation assay, which detects free GFP that is liberated upon the delivery of endogenous *ATG8* promoter-driven Atg8 with an N-terminal GFP tag (GFP-Atg8) to the vacuole and subsequent proteolysis of Atg8.
For Supplementary Fig. 5d and 5e, shown are the typical example of three biological independent replicates.
For Supplementary Fig. 5a-5c, the quantitative data represent means ± SE; n=3 biological independent experiments. Statistical significance was tested using two-sided Student's t-test. *n.s.*, no significance.

Supplementary Fig. 6

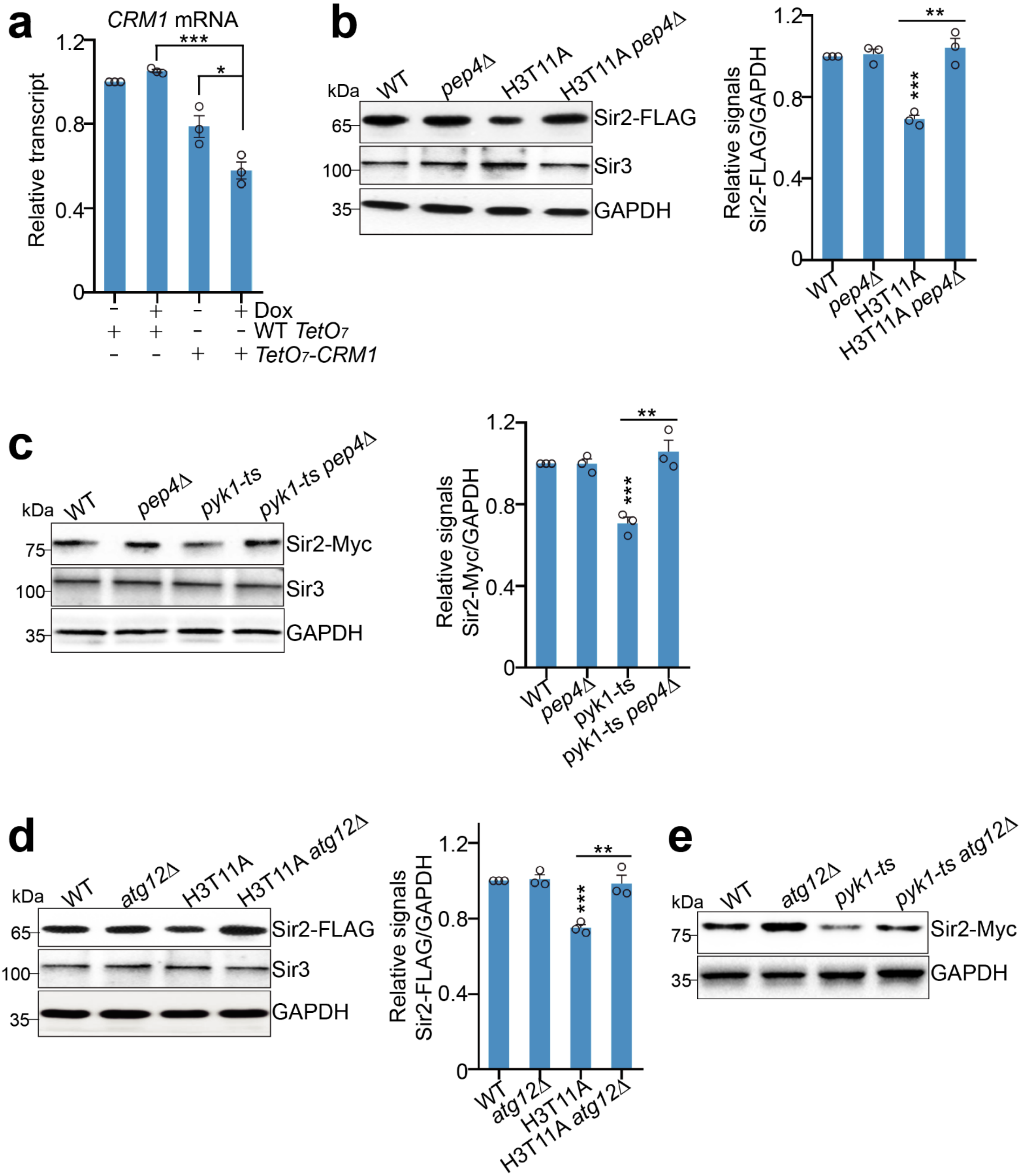

Supplementary Fig. 6 SESAME-catalyzed H3T11 phosphorylation prevents autophagy-mediated Sir2 degradation.

a, qRT-PCR analysis of *CRM1* expression in WT $TetO_7$ and $TetO_7$-*CRM1* treated with or without 6.25 μg/ml doxycycline (Dox) for 1.5 hrs.

b, Deletion of *PEP4* rescued the reduced Sir2 in H3T11A mutant.

c, Deletion of *PEP4* rescued the reduced Sir2 in *pyk1-ts* mutant.

d and **e**, Deletion of *ATG12* rescued the reduced Sir2 in H3T11A and *pyk1-ts* mutants.

For Supplementary Fig. 6c and 6e, these strains were treated at 37 °C for 2 hrs.

For Supplementary Fig. 6a-6d, the quantitative data represent means ± SE; n=3 biological independent experiments. For Supplementary Fig. 6e, shown are the typical example of three biological independent replicates. Statistical significance was tested using two-sided Student's t-test. *, $p<0.05$; **, $p<0.01$; ***, $p<0.001$.

Supplementary Fig. 7

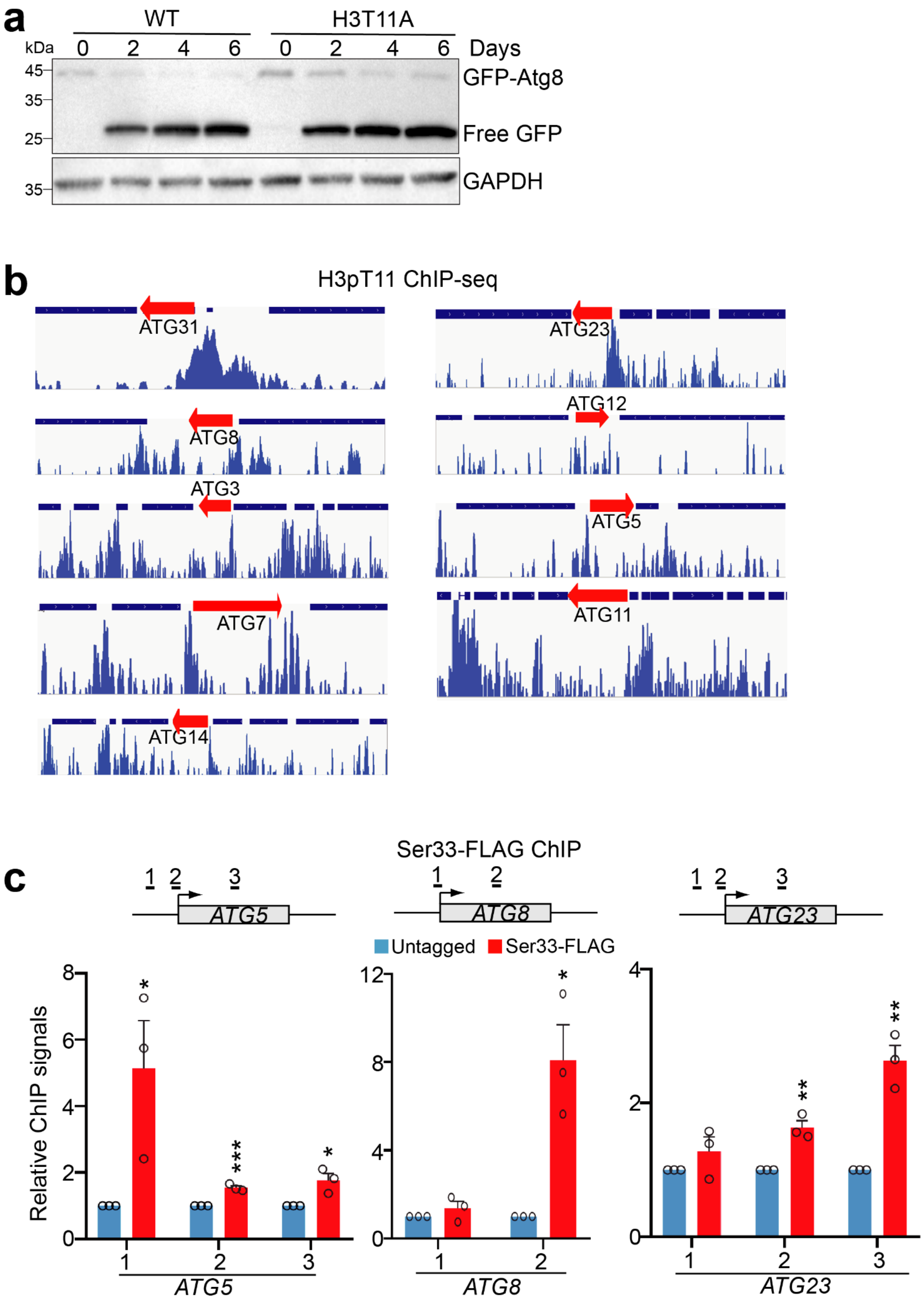

Supplementary Fig. 7 SESAME-catalyzed H3T11 phosphorylation prevents autophagy-mediated Sir2 degradation.

a, GFP-Atg8 processing assays were performed in WT and H3T11A mutant when aged for 0, 2, 4 and 6 days in YPD medium.

b, ChIP-seq tracks of the enrichment of H3pT11 at representative *ATG* genes. The location and orientation of *ATG* genes were labeled with red arrows.

c, ChIP analysis of Ser33 binding at *ATG5*, *ATG8,* and *ATG23* using the amplicons at each gene as indicated at the top panel.

For Supplementary Fig. 7a, shown are the typical example of three biological independent replicates. For Supplementary Fig. 7c, the quantitative data represent means ± SE; n=3 biological independent experiments. Statistical significance was tested using two-sided Student's t-test. *, $p<0.05$; **, $p<0.01$; ***, $p<0.001$.

Supplementary Fig. 8

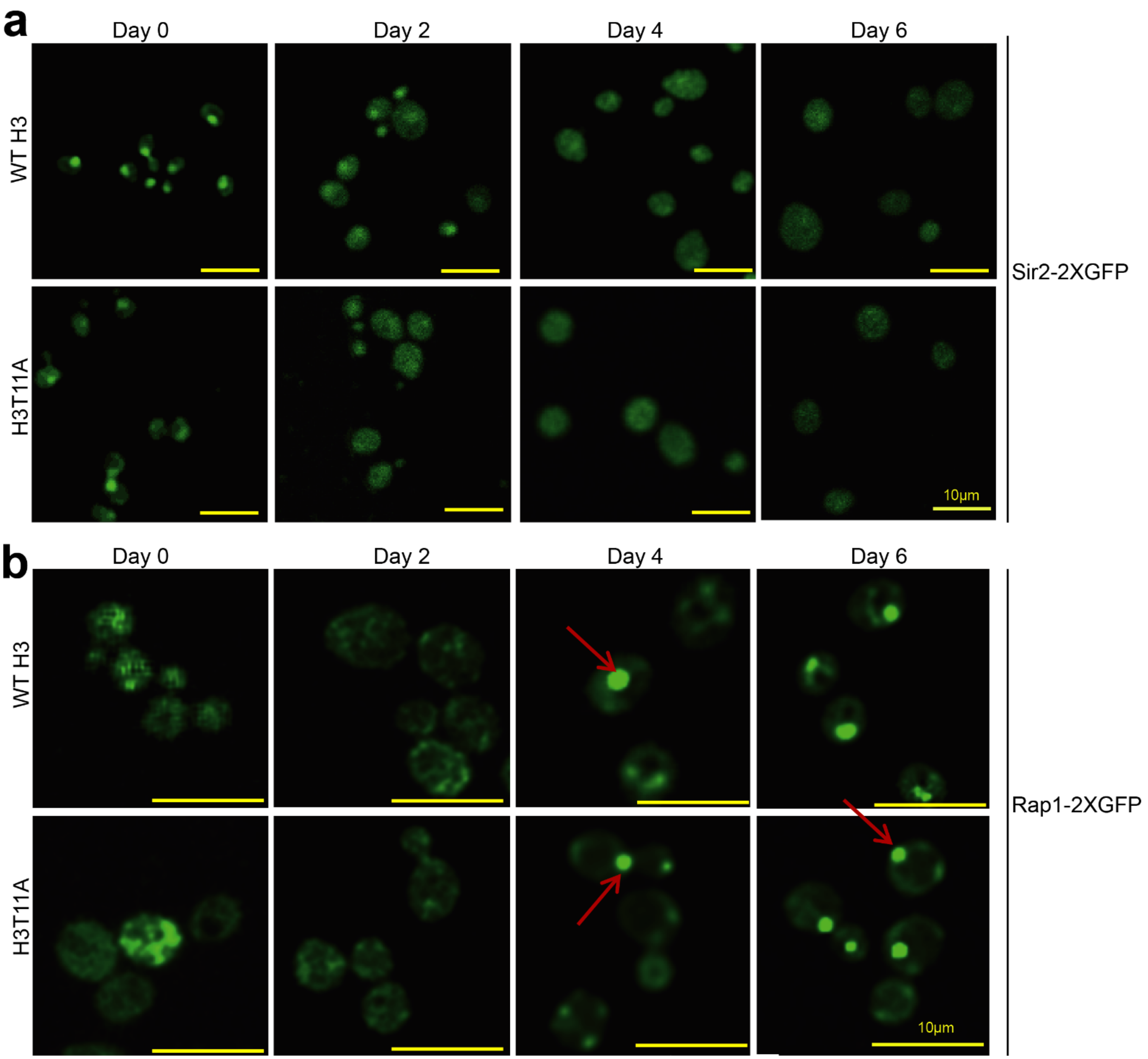

Supplementary Fig. 8 SESAME-catalyzed H3T11 phosphorylation has no effect on telomere hyperclustering.

a, Fluorescence images of Sir2-2xGFP in WT and H3T11A mutant when grown in YPD medium from day 0 to day 6. Scale, 10 μm.

b, Fluorescence images of Rap1-2xGFP in WT and H3T11A mutant when grown in YPD medium from day 0 to day 6. The telomere hyperclusters were indicated by red arrows. Scale, 10 μm.

For Supplementary Fig. 8a and 8b, the images shown are a typical example of three biological independent experiments.

Supplementary Table 1. List of strains used in this study

**Name**	**Parental Strain**	***Genotype***	**Source**
BY4741	BY4741	*MATa his3Δ1 leu2Δ0 met15Δ0 ura3Δ0*	Open Biosystems
sam1Δ	BY4741	*MATa his3Δ1 leu2Δ0 met15Δ0 ura3Δ0 sam1Δ::KAN*	Open Biosystems
sam2Δ	BY4741	*MATa his3Δ1 leu2Δ0 met15Δ0 ura3Δ0 sam2Δ::KAN*	Open Biosystems
ser33Δ	BY4741	*MATa his3Δ1 leu2Δ0 met15Δ0 ura3Δ0 ser33Δ::KAN*	Open Biosystems
shm2Δ	BY4741	*MATa his3Δ1 leu2Δ0 met15Δ0 ura3Δ0 shm2Δ::KAN*	Open Biosystems
sir2Δ	BY4741	*MATa his3Δ1 leu2Δ0 met15Δ0 ura3Δ0 sir2Δ::KAN*	Open Biosystems
sir4Δ	BY4741	*MATa his3Δ1 leu2Δ0 met15Δ0 ura3Δ0 sir4Δ::KAN*	Open Biosystems
sir3Δ	BY4741	*MATa his3Δ1 leu2Δ0 met15Δ0 ura3Δ0 sir3Δ::KAN*	Open Biosystems
Sir2-FLAG	BY4741	*MATa his3Δ1 leu2Δ0 met15Δ0 ura3Δ0 SIR2-3xFLAG::KAN*	In this study
Sir2-Myc	BY4741	*MATa his3Δ1 leu2Δ0 met15Δ0 ura3Δ0 SIR2-13xMYC::HIS3*	In this study
Sir2-Myc *atg2Δ*	BY4741	*MATa his3Δ1 leu2Δ0 met15Δ0 ura3Δ0 SIR2-13xMYC::HIS3 atg2Δ::KAN*	In this study
Sir2-Myc *atg12Δ*	BY4741	*MATa his3Δ1 leu2Δ0 met15Δ0 ura3Δ0 SIR2-13xMYC::HIS3 atg12Δ::KAN*	In this study
Sir2-Myc *pep4Δ*	BY4741	*MATa his3Δ1 leu2Δ0 met15Δ0 ura3Δ0 SIR2-13xMYC::HIS3 pep4Δ::KAN*	In this study
Sir2-Myc *atg5Δ*	BY4741	*MATa his3Δ1 leu2Δ0 met15Δ0 ura3Δ0 SIR2-13xMYC::HIS3 atg5Δ::KAN*	In this study
Sir2-Myc *atg7Δ*	BY4741	*MATa his3Δ1 leu2Δ0 met15Δ0 ura3Δ0 SIR2-13xMYC::HIS3 atg7Δ::KAN*	In this study
Sir2-Myc *atg9Δ*	BY4741	*MATa his3Δ1 leu2Δ0 met15Δ0 ura3Δ0 SIR2-13xMYC::HIS3 atg9Δ::KAN*	In this study
Sir2-FLAG *ser33Δ*	BY4741	*MATa his3Δ1 leu2Δ0 met15Δ0 ura3Δ0 SIR2-3xFLAG::KAN ser33Δ::URA3*	In this study
Ser33-FLAG	BY4741	*MATa his3Δ1 leu2Δ0 met15Δ0 ura3Δ0 SER33-3xFLAG::KAN*	In this study
Pyk1-FLAG	BY4741	*MATa his3Δ1 leu2Δ0 met15Δ0 ura3Δ0 PYK1-3xFLAG::KAN*	In this study
Sam1-FLAG	BY4741	*MATa his3Δ1 leu2Δ0 met15Δ0 ura3Δ0 SAM1-3xFLAG::KAN*	In this study
Pyk1-ts (cdc19-1)	S288C		Yeast Conditional Temperature Sensitive
Sir2-Myc *Pyk1-ts*	S288C	*cdc19-1 SIR2-13xMYC::HIS3*	In this study

Sir2-Myc<br>*Pyk1-ts atg2Δ*	S288C	*cdc19-1 SIR2-13xMYC::HIS3 atg2Δ::URA3*	In this study
Sir2-Myc<br>*Pyk1-ts atg12Δ*	S288C	*cdc19-1 SIR2-13xMYC::HIS3 atg12Δ::URA3*	In this study
Sir2-Myc<br>*Pyk1-ts pep4Δ*	S288C	*cdc19-1 SIR2-13xMYC::HIS3 pep4Δ::URA3*	In this study
Sir2-Myc<br>*atg12Δ*	BY4741	*MATa his3Δ1 leu2Δ0 met15Δ0 ura3Δ0, SIR2-13xMYC::HIS3 atg12Δ::URA3*	In this study
Sir2-Myc<br>*atg2Δ*	BY4741	*MATa his3Δ1 leu2Δ0 met15Δ0 ura3Δ0, SIR2-13xMYC::HIS3 atg2Δ::URA3*	In this study
Sir2-Myc<br>*pep4Δ*	BY4741	*MATa his3Δ1 leu2Δ0 met15Δ0 ura3Δ0, SIR2-13xMYC::HIS3 pep4Δ::URA3*	In this study
Wide type<br>(YBL)	S288C	*MATa his3Δ200 leu2Δ1 ura3-52, trp1Δ63 lys2-128δ (hht1-hhf1)Δ::LEU2, (hht2-hhf2)Δ::HIS3 Ty912 35- lacZ::his4 pDM18-HHT2-HHF2-TRP1*	Provided by Dr. Fred Winston
H3T11A<br>(YBL)	S288C	*MATa his3Δ200 leu2Δ1 ura3-52, trp1Δ63 lys2-128δ (hht1-hhf1)Δ::LEU2, (hht2-hhf2)Δ::HIS3 Ty912 35- lacZ::his4 pDM18-HHT2(T11A)-HHF2-TRP1*	In this study
H3T11D<br>(YBL)	S288C	*MATa his3Δ200 leu2Δ1 ura3-52, trp1Δ63 lys2-128δ (hht1-hhf1)Δ::LEU2, (hht2-hhf2)Δ::HIS3 Ty912 35- lacZ::his4 pDM18-HHT2(T11D)-HHF2-TRP1*	In this study
sam1Δ<br>(TEL)	UCC1369	*MATa ade2::hisG his3Δ200 leu2Δ0 lys2Δ0 met15Δ0 trp1Δ63 ura3Δ0 adh4::URA3-TEL-VIIL ADE2-TEL-VR , (hht2-hhf2)Δ::MET15 (hht1-hhf1)Δ::LEU2, pDM18-HHT2-HHF2-TRP1, sam1Δ::KAN*	In this study
shm2Δ<br>(TEL)	UCC1369	*MATa ade2::hisG his3Δ200 leu2Δ0 lys2Δ0 met15Δ0 trp1Δ63 ura3Δ0 adh4::URA3-TEL-VIIL ADE2-TEL-VR , (hht2-hhf2)Δ::MET15 (hht1-hhf1)Δ::LEU2, pDM18-HHT2-HHF2-TRP1, shm2Δ::KAN*	In this study
UCC1369	UCC1369	*MATa ade2::hisG his3Δ200 leu2Δ0 lys2Δ0 met15Δ0 trp1Δ63 ura3Δ0 adh4::URA3-TEL-VIIL ADE2-TEL-VR , (hht2-hhf2)Δ::MET15 (hht1-hhf1)Δ::LEU2, pDM18-HHT2-HHF2-TRP1*	Provided by Dr. Dan Gottschling
sir2Δ<br>(UCC1369)	UCC1369	*MATa ade2::hisG his3Δ200 leu2Δ0 lys2Δ0 met15Δ0 trp1Δ63 ura3Δ0 adh4::URA3-TEL-VIIL ADE2-TEL-VR , (hht2-hhf2)Δ::MET15 (hht1-hhf1)Δ::LEU2, pDM18-HHT2-HHF2-TRP1, sir2Δ::KAN*	In this study
H3T11A<br>(UCC1369)	UCC1369	*MATa ade2::hisG his3Δ200 leu2Δ0 lys2Δ0 met15Δ0 trp1Δ63 ura3Δ0 adh4::URA3-TEL-VIIL ADE2-TEL-VR , (hht2-hhf2)Δ::MET15 (hht1-hhf1)Δ::LEU2, pDM18-HHT2(T11A)-HHF2-TRP1*	In this study
sam1Δ<br>H3T11A<br>(UCC1369)	UCC1369	*MATa ade2::hisG his3Δ200 leu2Δ0 lys2Δ0 met15Δ0 trp1Δ63 ura3Δ0 adh4::URA3-TEL-VIIL ADE2-TEL-VR , (hht2-hhf2)Δ::MET15 (hht1-hhf1)Δ::LEU2, pDM18-HHT2(T11A)-HHF2-TRP1 sam1Δ::KAN*	In this study
shm2Δ<br>H3T11A<br>(UCC1369)	UCC1369	*MATa ade2::hisG his3Δ200 leu2Δ0 lys2Δ0 met15Δ0 trp1Δ63 ura3Δ0 adh4::URA3-TEL-VIIL ADE2-TEL-VR , (hht2-hhf2)Δ::MET15 (hht1-hhf1)Δ::LEU2, pDM18-HHT2(T11A)-HHF2-TRP1 shm2Δ::KAN*	In this study

Sir2-FLAG WT H3 (YBL)	S288C	*MATa his3Δ200 leu2Δ1 ura3-52, trp1Δ63 lys2-128δ (hht1-hhf1)Δ::LEU2, (hht2-hhf2)Δ::HIS3 Ty912 35- lacZ::his4 pDM18-HHT2-HHF2-TRP1 SIR2-3xFLAG::KAN*	In this study
Sir2-FLAG H3T11A (YBL)	S288C	*MATa his3Δ200 leu2Δ1 ura3-52, trp1Δ63 lys2-128δ (hht1-hhf1)Δ::LEU2, (hht2-hhf2)Δ::HIS3 Ty912 35- lacZ::his4 pDM18-HHT2(T11A)-HHF2-TRP1 SIR2-3xFLAG::KAN*	In this study
Sir2-FLAG H3T11D (YBL)	S288C	*MATa his3Δ200 leu2Δ1 ura3-52, trp1Δ63 lys2-128δ (hht1-hhf1)Δ::LEU2, (hht2-hhf2)Δ::HIS3 Ty912 35- lacZ::his4, pDM18-HHT2(T11D)-HHF2-TRP1 SIR2-3xFLAG::KAN*	In this study
Sir3-FLAG H3T11A (YBL)	S288C	*MATa his3Δ200 leu2Δ1 ura3-52, trp1Δ63 lys2-128δ (hht1-hhf1)Δ::LEU2, (hht2-hhf2)Δ::HIS3 Ty912 35- lacZ::his4 pDM18-HHT2(T11A)-HHF2-TRP1 SIR3-3xFLAG::KAN*	In this study
Sir3-FLAG (YBL)	S288C	*MATa his3Δ200 leu2Δ1 ura3-52, trp1Δ63 lys2-128δ (hht1-hhf1)Δ::LEU2, (hht2-hhf2)Δ::HIS3 Ty912 35- lacZ::his4 pDM18-HHT2-HHF2-TRP1 SIR3-3xFLAG::KAN*	In this study
Sir4-FLAG (YBL)	S288C	*MATa his3Δ200 leu2Δ1 ura3-52, trp1Δ63 lys2-128δ (hht1-hhf1)Δ::LEU2, (hht2-hhf2)Δ::HIS3 Ty912 35- lacZ::his4 pDM18-HHT2-HHF2-TRP1 SIR4-3xFLAG::KAN*	In this study
Sir4-FLAG H3T11A (YBL)	S288C	*MATa his3Δ200 leu2Δ1 ura3-52, trp1Δ63 lys2-128δ (hht1-hhf1)Δ::LEU2, (hht2-hhf2)Δ::HIS3 Ty912 35- lacZ::his4 pDM18-HHT2(T11A)-HHF2-TRP1 SIR4-3xFLAG::KAN*	In this study
Sir2-FLAG *atg2Δ* (YBL)	S288C	*MATa his3Δ200 leu2Δ1 ura3-52, trp1Δ63 lys2-128δ (hht1-hhf1)Δ::LEU2, (hht2-hhf2)Δ::HIS3 Ty912 35- lacZ::his4, pDM18-HHT2-HHF2-TRP1, SIR2-3xFLAG::KAN atg2Δ::URA3*	In this study
Sir2-FLAG H3T11A *atg2Δ* (YBL)	S288C	*MATa his3Δ200 leu2Δ1 ura3-52, trp1Δ63 lys2-128δ (hht1-hhf1)Δ::LEU2, (hht2-hhf2)Δ::HIS3 Ty912 35- lacZ::his4, pDM18-HHT2(T11A)-HHF2-TRP1, SIR2-3xFLAG::KAN, atg2Δ::URA3*	In this study
Sir2-FLAG *atg12Δ* (YBL)	S288C	*MATa his3Δ200 leu2Δ1 ura3-52, trp1Δ63 lys2-128δ (hht1-hhf1)Δ::LEU2, (hht2-hhf2)Δ::HIS3 Ty912 35- lacZ::his4, pDM18-HHT2-HHF2-TRP1, SIR2-3xFLAG::KAN , atg12Δ::URA3*	In this study
Sir2-FLAG H3T11A *atg12Δ* (YBL)	S288C	*MATa his3Δ200 leu2Δ1 ura3-52, trp1Δ63 lys2-128δ (hht1-hhf1)Δ::LEU2, (hht2-hhf2)Δ::HIS3 Ty912 35- lacZ::his4, pDM18-HHT2(T11A)-HHF2-TRP1, SIR2-3xFLAG::KAN, atg12Δ::URA3*	In this study
Sir2-FLAG *pep4Δ* (YBL)	S288C	*MATa his3Δ200 leu2Δ1 ura3-52, trp1Δ63 lys2-128δ (hht1-hhf1)Δ::LEU2, (hht2-hhf2)Δ::HIS3 Ty912 35- lacZ::his4, pDM18-HHT2-HHF2-TRP1, SIR2-3xFLAG::KAN, pep4Δ::URA3*	In this study
Sir2-FLAG H3T11A *pep4Δ* (YBL)	S288C	*MATa his3Δ200 leu2Δ1 ura3-52, trp1Δ63 lys2-128δ (hht1-hhf1)Δ::LEU2, (hht2-hhf2)Δ::HIS3 Ty912 35- lacZ::his4, pDM18-HHT2(T11A)-HHF2-TRP1, SIR2-3xFLAG::KAN, pep4Δ::URA3*	In this study
ATG8p-GFP-Atg8	BY4741	*MATa his3Δ1 leu2Δ0 met15Δ0 ura3Δ0, ATG8p-GFP-ATG8-URA3*	In this study
Sir2-FLAG WT H3(YBL)/	S288C	*MATa his3Δ200 leu2Δ1 ura3-52, trp1Δ63 lys2-128δ (hht1-hhf1)Δ::LEU2, (hht2-hhf2)Δ::HIS3 Ty912 35- lacZ::his4,*	In this study

ATG8p-GFP-Atg8		*pDM18-HHT2-HHF2-TRP1 SIR2-3xFLAG::KAN, ATG8p-GFP-ATG8-URA3*	
Sir2-FLAG H3T11A (YBL)/ATG8p-GFP-Atg8	S288C	*MATa his3Δ200 leu2Δ1 ura3-52, trp1Δ63 lys2-128δ (hht1-hhf1)Δ::LEU2, (hht2-hhf2)Δ::HIS3, Ty912 35- lacZ::his4, pDM18-HHT2(T11A)-HHF2-TRP1, SIR2-3xFLAG::KAN, ATG8p-GFP-ATG8-URA3*	In this study
Pyk1-ts/ATG8p-GFP-Atg8	S288C	*cdc19-1 ATG8p-GFP-ATG8-URA3*	In this study
Sir2-Myc *sir4Δ*	BY4741	*MATa his3Δ1 leu2Δ0 met15Δ0 ura3Δ0 SIR2-13xMYC::HIS3 sir4Δ::KAN*	In this study
Sir2-Myc *sir3Δ*	BY4741	*MATa his3Δ1 leu2Δ0 met15Δ0 ura3Δ0 SIR2-13xMYC::HIS3 sir3Δ:: KAN*	In this study
Sir2-Myc R1158	R1158	*MATa URA3::CMV-tTA his3-1 leu2-0 met15-0 ura3-1 URA3::CMV-tTA SIR2-13xMYC::HIS3*	In this study
Sir2-Myc *TetO$_7$-CRM1*	R1158	*MATa URA3::CMV-tTA his3-1 leu2-0 met15-0 ura3-1 pCRM1::kanR-tet07-TATA URA3::CMV-tTA, SIR2-13xMYC::HIS3*	In this study
Sir2-GFP R1158	R1158	*MATa URA3::CMV-tTA his3-1 leu2-0 met15-0 ura3-1 SIR2-2xGFP::KAN*	In this study
Sir2-GFP *TetO$_7$-CRM1*	R1158	*MATa URA3::CMV-tTA his3-1 leu2-0 met15-0 ura3-1 pCRM1::kanR-tet07-TATA URA3::CMV-tTA, SIR2-2xGFP::KAN*	In this study
WT (Empty vector)	S288C	*MATa ade2::hisG his3Δ200 leu2Δ0 lys2Δ0 met15Δ0 trp1Δ63 ura3Δ0 adh4::URA3-TEL-VIIL ADE2-TEL-VR , (hht2-hhf2)Δ::MET15 (hht1-hhf1)Δ::LEU2, pDM18-HHT2-HHF2-TRP1, pTEF-HIS3*	In this study
WT (pTEFpro-SIR2)	S288C	*MATa ade2::hisG his3Δ200 leu2Δ0 lys2Δ0 met15Δ0 trp1Δ63 ura3Δ0 adh4::URA3-TEL-VIIL ADE2-TEL-VR , (hht2-hhf2)Δ::MET15 (hht1-hhf1)Δ::LEU2, pDM18-HHT2-HHF2-TRP1, pTEF-SIR2-HIS3*	In this study
H3T11A (Empty vector)	S288C	*MATa ade2::hisG his3Δ200 leu2Δ0 lys2Δ0 met15Δ0 trp1Δ63 ura3Δ0 adh4::URA3-TEL-VIIL ADE2-TEL-VR , (hht2-hhf2)Δ::MET15 (hht1-hhf1)Δ::LEU2, pDM18-HHT2(T11A)-HHF2-TRP1, pTEF-HIS3*	In this study
H3T11A (pTEFpro-SIR2)	S288C	*MATa ade2::hisG his3Δ200 leu2Δ0 lys2Δ0 met15Δ0 trp1Δ63 ura3Δ0 adh4::URA3-TEL-VIIL ADE2-TEL-VR , (hht2-hhf2)Δ::MET15 (hht1-hhf1)Δ::LEU2, pDM18-HHT2(T11A)-HHF2-TRP1, pTEF-SIR2-HIS3*	In this study
Rap1-GFP WT H3	S288C	*MATa his3Δ200 leu2Δ0 lys2Δ0 trp1Δ63 ura3Δ0 met15Δ0 can1::MFA1pr-HIS3 hht1-hhf1::NatMX4 hht2-hhf2::[HHTS-HHFS]*-URA3 RAP1-2xGFP::KAN*	In this study
Rap1-GFP H3T11A	S288C	*MATa his3Δ200 leu2Δ0 lys2Δ0 trp1Δ63 ura3Δ0 met15Δ0 can1::MFA1pr-HIS3 hht1-hhf1::NatMX4 hht2-hhf2::[HHTS(T11A)-HHFS]*-URA3 RAP1-2xGFP::KAN*	In this study
Ser33-FLAG *set1Δ*	BY4741	*MATa his3Δ1 leu2Δ0 met15Δ0 ura3Δ0 SER33-3xFLAG::KAN set1Δ::HIS3*	In this study
Pyk1-FLAG *set1Δ*	BY4741	*MATa his3Δ1 leu2Δ0 met15Δ0 ura3Δ0 PYK1-3xFLAG::KAN set1Δ::HIS3*	In this study
Sam1-FLAG *set1Δ*	BY4741	*MATa his3Δ1 leu2Δ0 met15Δ0 ura3Δ0 SAM1-3xFLAG::KAN set1Δ::HIS3*	In this study

Sir2-FLAG *set1*Δ	BY4741	*MATa his3Δ1 leu2Δ0 met15Δ0 ura3Δ0 SIR2-3xFLAG::KAN set1Δ::HIS3*	In this study
Sir2-FLAG H3K4A (YBL)	S288C	*MATa his3Δ200 leu2Δ1 ura3-52, trp1Δ63 lys2-128δ (hht1-hhf1)Δ::LEU2, (hht2-hhf2)Δ::HIS3 Ty912 35- lacZ::his4 pDM18-HHT2(K4A)-HHF2-TRP1 SIR2-3xFLAG::KAN*	In this study
Sir2-FLAG WT H3 (Integrated H3/H4)	S288C	*MATa his3Δ200 leu2Δ0 lys2Δ0 trp1Δ63 ura3Δ0 met15Δ0 can1::MFA1pr-HIS3 hht1-hhf1::NatMX4 hht2-hhf2::[HHTS-HHFS]*-URA3 SIR2-3xFLAG::KAN*	In this study
Sir2-FLAG H3T11A (Integrated H3/H4)	S288C	*MATa his3Δ200 leu2Δ0 lys2Δ0 trp1Δ63 ura3Δ0 met15Δ0 can1::MFA1pr-HIS3 hht1-hhf1::NatMX4 hht2-hhf2::[HHTS(T11A)-HHFS]*-URA3 SIR2-3xFLAG::KAN*	In this study

Supplementary Table 2. List of primers used in this study

**Gene name**	**Sequence**
ChIP	
TEL VIR 1kb	GTTATGTTAGAGATAACTGTGAG
	GCTTGTTAACTCTCCGACAG
TEL VIR 2.5kb	GCAATGAATCTTCGGTGCTTGG
	CCATACCAATATCAACTTCACGG
TEL VIR 5kb	CCCCGCCTTTGAAGATTGTCCC
	CGAGACCCACTTGTATTCTTAGTGC
TEL VIR 7.5kb	CCTCTATAGGACCTGTCTCATGG
	GGAAGTCTACACTAATAGCTATG
TEL VIR 15kb	GCGCAATATATAGCAGAAGAGC
	CAATTCGTCGATAAAGTGC
TEL VIR-L	CCACACCCACACCCTAATAC
	GGGTAATGGAGGGTAAGTTGAG
TEL VIR-R	AGGATTATAGGTAAATGGCAAGGG
	GACCCAGTCCTCATTTCCATC
TEL VIII-L	TTACCCCATCTAAAGTGCCG
	ACAACCACACCTCCGAAATC
TEL VIII -R	CACACCCACACTTTTCACATC
	GCTATGTAGAAGTGCTGTAGGG
TEL XII-L	ATCTACCTCTACTCTCGCTGTC
	CGTACATGAGGGCTATTTAGGG
TEL XII-R	TTACCCCATCTAAAGTGCCG
	ACAACCACACCTCCGAAATC
ATG5-1	ACCAGGTAAAGGATGTTCTCAC
	TGCGATGGGAATGATAGTTGG
ATG5-2	AACTGTGGCAAGAACATCGC
	AGGCGTTATGTTTCTCATCGC

ATG5-3	AGCATGCTCAGAAGTGCGAA
	ACATCATAGGTTTCCTATCTCCGT
ATG8-1	ACCTTACCGTAGGGCAATTTG
	CCCGTCCTTATCCTTGTGTTC
ATG8-2	ACCCGTGAAATCATAGCACAT
	ACCAAATATTTTTGCCGCCG
ATG23-1	CTCCATAGCGAAAGTACCACTG
	CGACCCTTTATGGCTTTTGTG
ATG23-2	ATAGAACACAGAGAACGTCTG
	CCATTCCTTAGCCGGTTCTCC
ATG23-3	AGTTCAAGTGATTATTTTCGTTTTCTTGT
	CTTCTTCACTTTATTTTGTTACCTTATAGAA
PHO11	GACAAAATCGGAACTCAAACGG
	TCTTTCACCGTGTCTACCAAC
COS8	CCGTTCTACCTCAAGATGTTTTCCG
	CCAGGAACAGGACAAGAAGTGAAAC
YFR057W	TGATATTTGGACCTACTAGTGTCTATAG
	GCTTGGCGGTGTCTTTAATG
SEO1	GGTTTAGAGGGATGGAGATGG
	TCCTCGCCAACCTAATTTCATC
YCR106W	CGCCTTAGGGTTATTATACAATGC
	CGCTCTCAAAGAGTGAAATGTCC
SOR1	GTAGTTCTAGAGAAAGTCGGCG
	GGCGCCTTCAATATGTACTTACC
RDN58-1	GCGAAATGCGATACGTAATGTG
	GGCGCAATGTGCGTTCA
HML-α-1	TCAATATTATTCGACCACTCAAGAAAG
	CGCTATCCTGTGAATTTGGATTT
YJR011C	CCGCTTATTATTGGATAGTTGTGAG
	TCGACATTTAATCTGTTTAATTCCTGG
PYK1	GCTACGTAAATGTGTTCCGCAC
	GGTACCTAGCATCATATGGGAAGG
TEL II 0.2kb	GTTGATTGGCAAAGCTCCAC
	CATGGCATCCACCAAAATATGG
TEL II 2.8kb	AGAGAAAGAGCGGAGAAACAG
	GCCTTAAAATGCCCTCTGTTG
TEL II 5.7kb	GAAATTGGAAGCGTATGACTTCG
	CCACTACCCTGATCTGCAATC
TEL II 9.7kb	ACCTTGATTTTGACGCTTTTGAG
	TGAGATCTTCCGACCGTTTG
qRT-PCR	
ACTIN	CTGTCGAGAGATTTCTCTTTTACC
	GCCCCTATTTATTCCAATAATATCG
PHO11	GACAAAATCGGAACTCAAACGG
	TCTTTCACCGTGTCTACCAAC
YFR057W	TGATATTTGGACCTACTAGTGTCTATAG
	GCTTGGCGGTGTCTTTAATG

COS8	CCGTTCTACCTCAAGATGTTTTCCG
	CCAGGAACAGGACAAGAAGTGAAAC
YCR106W	CGCCTTAGGGTTATTATACAATGC
	CGCTCTCAAAGAGTGAAATGTCC
SOR1	GTAGTTCTAGAGAAAGTCGGCG
	GGCGCCTTCAATATGTACTTACC
SEO1	GGTTTAGAGGGATGGAGATGG
	TCCTCGCCAACCTAATTTCATC
SIR2	CCTCGAACTTCCACTATGCC
	TGAGAACGCCATATGAGTTAAGG
URA3	CAGAATTGTCATGCAAGGGC
	GTAACCTTCATCTCTTCCACCC
ADE2	TCTCTGTCGCTCAAAAGTTGG
	GTCACTTCAAATGGAACGCC
ATG3	TTGCTAGATAAGGTTCGTGTGG
	CAAGTATTGGTCTACCCGTAACG
ATG5	ACCAGGTAAAGGATGTTCTCAC
	TGCGATGGGAATGATAGTTGG
ATG7	TCCCTGTTTTCAAAGACCCTC
	GAGAACAGCCCTTTTAAACTCG
ATG8	ACCTTACCGTAGGGCAATTTG
	CCCGTCCTTATCCTTGTGTTC
ATG11	AACTCCCCTAATTCCAACGAC
	AACGCGAAAGATCTACGTCTG
ATG12	GGAACGGCAATGGAAAGATC
	TCAACTTGCTGGTCGACAG
ATG14	CAAGATGAAGTGTAGGTCCGTC
	CATGAGGTCCTGTGACTGTTG
ATG29	CTATTAAATGTATCCGCAAGCCC
	CGCCTCATTTGTTACTTCTGTC
ATG23	CTCCATAGCGAAAGTACCACTG
	CGACCCTTTATGGCTTTTGTG
ATG31	AGCGTTGACAGTGATAGATTCAG
	GGAGACAGATCGCAAAATTGTG
ATG32	CAGCATACGAACACCAAACAG
	TGATTGTGTCGCTAGAGGAATC
SIR3	ATGGAAATATACCGTCTCAGCG
	AATCTGTAGGTGATGTGAGCG
SIR4	CAGATGCCAAAATTATGCCTAAGG
	TCTGGTAGGGTTTTCGTTTCC
CRM1	CGCCGAAGTGCTAAATTGTATG
	ACGATGCTCTGGATATTCTGTG
Primers for construct preparation	
pTEFpro-SIR2	TTTCTAGAACTAGTGGATCCATGACCATCCCACATATG AAA
	AGAAAACGTGAAACAAGCCCCAAATATGCATGTCTGG TTAACTATAGGGCGAATTGGGT

Supplementary Table 3. List of antibodies used in this study

Name	Company	Cat. No
Anti-Histone H3	Cell Signaling Technology	9715S
Anti-Histone H3	Abcam	ab1791
Anti-H3T11 phosphorylation	Abcam	ab5168
Anti-Flag M2	Sigma-Aldrich	F1804-1MG
Anti-H4K16 acetylation	EMD Millipore	07-329
Anti-Sir2	Santa Cruz Biotechnology	sc-6667
Anti-Sir3	Santa Cruz Biotechnology	sc-101612
Anti-GAPDH	proteintech	10494-1-AP
Anti-GFP	proteintech	66002-1-1g
Anti-Myc	proteintech	60003-2-1g
goat polyclonal anti-mouse IgG	proteintech	SA00001-1
goat polyclonal anti-rabbit IgG	proteintech	SA00001-2

References

1. Mei, Q. *et al.* Set1-catalyzed H3K4 trimethylation antagonizes the HIR/Asf1/Rtt106 repressor complex to promote histone gene expression and chronological life span. *Nucleic Acids Res.* **47**, 3434-3449 (2019).
